# Thirty novel sequence variants impacting human intracranial volume

**DOI:** 10.1093/braincomms/fcac271

**Published:** 2022-10-25

**Authors:** Muhammad Sulaman Nawaz, Gudmundur Einarsson, Mariana Bustamante, Rosa S Gisladottir, G Bragi Walters, Gudrun A Jonsdottir, Astros Th Skuladottir, Gyda Bjornsdottir, Sigurdur H Magnusson, Bergrun Asbjornsdottir, Unnur Unnsteinsdottir, Engilbert Sigurdsson, Palmi V Jonsson, Vala Kolbrun Palmadottir, Sigurjon A Gudjonsson, Gisli H Halldorsson, Egil Ferkingstad, Ingileif Jonsdottir, Gudmar Thorleifsson, Hilma Holm, Unnur Thorsteinsdottir, Patrick Sulem, Daniel F Gudbjartsson, Hreinn Stefansson, Thorgeir E Thorgeirsson, Magnus O Ulfarsson, Kari Stefansson

**Affiliations:** deCODE genetics/Amgen Inc., Sturlugata 8, 102 Reykjavik, Iceland; Faculty of Medicine, School of Health Sciences, University of Iceland, Vatnsmyrarvegur 16, 101 Reykjavik, Iceland; deCODE genetics/Amgen Inc., Sturlugata 8, 102 Reykjavik, Iceland; deCODE genetics/Amgen Inc., Sturlugata 8, 102 Reykjavik, Iceland; deCODE genetics/Amgen Inc., Sturlugata 8, 102 Reykjavik, Iceland; School of Humanities, University of Iceland, Saemundargata 2, 102 Reykjavik, Iceland; deCODE genetics/Amgen Inc., Sturlugata 8, 102 Reykjavik, Iceland; Faculty of Medicine, School of Health Sciences, University of Iceland, Vatnsmyrarvegur 16, 101 Reykjavik, Iceland; deCODE genetics/Amgen Inc., Sturlugata 8, 102 Reykjavik, Iceland; deCODE genetics/Amgen Inc., Sturlugata 8, 102 Reykjavik, Iceland; deCODE genetics/Amgen Inc., Sturlugata 8, 102 Reykjavik, Iceland; deCODE genetics/Amgen Inc., Sturlugata 8, 102 Reykjavik, Iceland; deCODE genetics/Amgen Inc., Sturlugata 8, 102 Reykjavik, Iceland; deCODE genetics/Amgen Inc., Sturlugata 8, 102 Reykjavik, Iceland; Faculty of Medicine, School of Health Sciences, University of Iceland, Vatnsmyrarvegur 16, 101 Reykjavik, Iceland; Department of Psychiatry, Landspitali-National University Hospital, Hringbraut 101, 101 Reykjavik, Iceland; Faculty of Medicine, School of Health Sciences, University of Iceland, Vatnsmyrarvegur 16, 101 Reykjavik, Iceland; Department of Geriatric Medicine, Landspitali University Hospital, Hringbraut 101, 101 Reykjavik, Iceland; Department of Internal Medicine, Landspitali University Hospital, Hringbraut 101, 101 Reykjavik, Iceland; deCODE genetics/Amgen Inc., Sturlugata 8, 102 Reykjavik, Iceland; deCODE genetics/Amgen Inc., Sturlugata 8, 102 Reykjavik, Iceland; School of Engineering and Natural Sciences, University of Iceland, Taeknigardur, Dunhagi 5, 107 Reykjavik, Iceland; deCODE genetics/Amgen Inc., Sturlugata 8, 102 Reykjavik, Iceland; deCODE genetics/Amgen Inc., Sturlugata 8, 102 Reykjavik, Iceland; deCODE genetics/Amgen Inc., Sturlugata 8, 102 Reykjavik, Iceland; deCODE genetics/Amgen Inc., Sturlugata 8, 102 Reykjavik, Iceland; deCODE genetics/Amgen Inc., Sturlugata 8, 102 Reykjavik, Iceland; deCODE genetics/Amgen Inc., Sturlugata 8, 102 Reykjavik, Iceland; deCODE genetics/Amgen Inc., Sturlugata 8, 102 Reykjavik, Iceland; deCODE genetics/Amgen Inc., Sturlugata 8, 102 Reykjavik, Iceland; deCODE genetics/Amgen Inc., Sturlugata 8, 102 Reykjavik, Iceland; deCODE genetics/Amgen Inc., Sturlugata 8, 102 Reykjavik, Iceland; Faculty of Electrical and Computer Engineering, University of Iceland, Taeknigardur, Dunhagi 5, 107 Reykjavik, Iceland; deCODE genetics/Amgen Inc., Sturlugata 8, 102 Reykjavik, Iceland; Faculty of Medicine, School of Health Sciences, University of Iceland, Vatnsmyrarvegur 16, 101 Reykjavik, Iceland

**Keywords:** intracranial volume, genome-wide association study, Mendelian randomization, genetic correlation, brain structure

## Abstract

Intracranial volume, measured through magnetic resonance imaging and/or estimated from head circumference, is heritable and correlates with cognitive traits and several neurological disorders. We performed a genome-wide association study meta-analysis of intracranial volume (*n* = 79 174) and found 64 associating sequence variants explaining 5.0% of its variance. We used coding variation, transcript and protein levels, to uncover 12 genes likely mediating the effect of these variants, including *GLI3* and *CDK6* that affect cranial synostosis and microcephaly, respectively. Intracranial volume correlates genetically with volumes of cortical and sub-cortical regions, cognition, learning, neonatal and neurological traits. Parkinson’s disease cases have greater and attention deficit hyperactivity disorder cases smaller intracranial volume than controls. Our Mendelian randomization studies indicate that intracranial volume associated variants either increase the risk of Parkinson’s disease and decrease the risk of attention deficit hyperactivity disorder and neuroticism or correlate closely with a confounder.

## Teaser

A genetic association study of 79 174 intracranial volumes, identified 64 variants, explaining 5.0% of the trait variance. Intracranial volume correlates genetically with cortical and sub-cortical volumes, cognition, learning, neonatal and neurological traits. Parkinson’s cases have greater, and attention-deficit hyperactivity disorder cases have smaller intracranial volumes, respectively.

## Graphical Abstract

**Graphical abstract:**
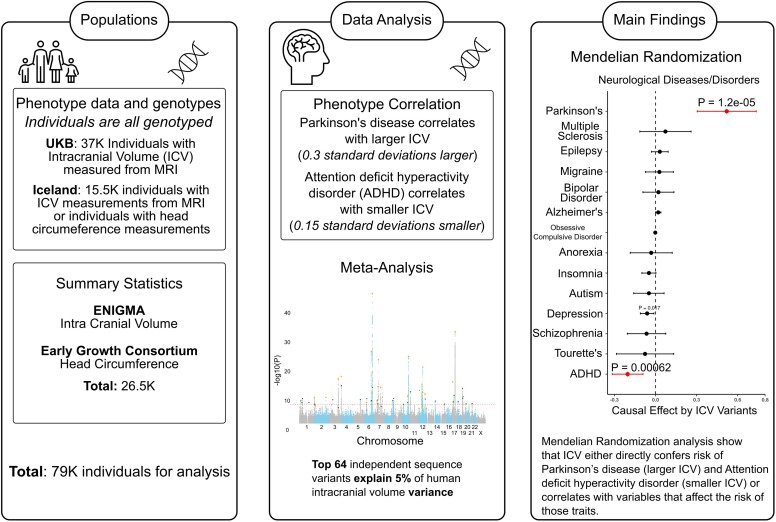

## Introduction

Overall intracranial volume (ICV) can be measured with CT or MRI and/or estimated through head circumference (HC) measurements. ICV and HC are heritable^1,2^ and highly correlated, both genetically (*rg* = 0.91)^3^ and phenotypically (*r* = 0.73).^4^ Studying HC, which is easily measured, allows for increased sample size for association studies. By finding sequence variants correlating with ICV, we can study the biological relationships between ICV and brain function and dysfunction. Variations in brain structure are associated with several neurological disorders.^5–7^ Genetically, attention deficit hyperactivity disorder (ADHD) is negatively correlated with ICV (*rg* = −0.23)^8^ while Parkinson's disease positively correlates with ICV (*rg* = 0.35).^9^ Nalls *et al*.^9^ reported via Mendelian randomization (MR) analysis that educational attainment increases risk of Parkinson’s disease. However, they did not study the effect of ICV on Parkinson’s disease.

Three meta-analyses of genome-wide association study (GWAS) results have previously uncovered sequence variants associating with ICV^10,11^ and cortical structures.^12^ The largest reported GWAS for ICV (*n* = 47 316)^8^ uncovered 18 variants, including an inversion polymorphism at 17q21.31, first described in the Icelandic population.^13^ This inversion polymorphism associates with many phenotypes, including personality traits,^14^ cognition,^15^ handedness,^16^ brain age^17^ and Parkinson’s disease.^9^ Rare variants have also been associated with HC and many of these are single nucleotide polymorphisms (SNPs) conferring risk of neurodevelopmental disorders, intellectual disability, microcephaly and macrocephaly.^18–25^ It has also been shown that variants in the sequence of the germline genome can impact the white^7^ or the grey matter^26^ volumes. Furthermore, several rare recurrent copy number variants have been associated with ICV and/or regional volumes and for some the effects are dosage dependent.^5,6,27^ ICV and cortical structures are genetically correlated with cognitive functions,^10^ Parkinson’s disease, insomnia, depression, neuroticism and ADHD.^12^ Environmental factors, including infections during pregnancy, may impact ICV and are known causes of microcephaly.^28^

Here, we present a GWAS meta-analysis of ICV (*n* = 79 174 Europeans), where we study rare and common sequence variants and find 64 variants, of which 30 are novel. Ten of the associated variants are coding, five are *cis*/*trans*-pQTLs, and several are *cis*-eQTLs for a single or multiple genes. We conclude from our MR analysis that ICV either directly affects neurological traits or correlates with variables that affect the risk of those traits.

## Methods

### Phenotyping and cohorts included in the discovery meta-analysis

#### ICV data

The ICVs were either determined from HC or ICV data from the samples of the participants. These measurements were adjusted for known confounders (e.g. height, sex, age, age^2^, sex × age^2^), and the residuals were rank transformed and inverse normalized to use for association studies.

#### Iceland ICV and HC

In Iceland, the ICV data of 1392 participants were extracted from MRI acquisitions as described earlier.^5,6^ These subjects participated in the various projects at deCODE genetics/Amgen. The ICV data were adjusted for known confounders,^5,6^ the residuals were rank transformed, and inverse normalized.

Additionally, we used manual HC measurements from 12 506 adults, and 1599 subjects measured in childhood who participated in various research projects at deCODE genetics, mostly as adults. At deCODE's recruitment centre, the HC measurements were performed as a part of a comprehensive phenotyping of a general population sample (the deCODE health study). For adults, the HC measurements were performed manually using a measuring tape, while the participant remained in a seated position, and each measurement was repeated three times, documenting only the largest value. Thus, the largest possible circumference was measured, from the most prominent part of the forehead above the ears to the most prominent part of the crown. While for children, HC measurements were performed during routine development assessment by Icelandic healthcare staff, using a measuring tape, with the child lying down, from the most prominent part of the forehead above the ears to the most prominent part of the crown.

The HC measures were also adjusted for known confounders (height, sex, age, age^2^ and sex × age^2^) and the residuals were rank transformed, and inverse normalized. The Pearson correlation between the ICV and HC measurements is high (*N*_ICV + HC data_ = 1392, *r* = 0.69, *P* = 6.27 × 10^−92^) as close to reported correlation (*r* = 0.73, *P* < 0.01).^4^ The residual of the inverse normalized, rank transformed and adjusted data of ICV and HC were combined (used ICV data where both ICV and HC were available) and used as a quantitative trait to run for association analysis. All of the participants (or their parents/guardian in case of minor) of the study gave written informed consent, in accordance with the declaration of Helsinki, and study was approved by the Icelandic Data Protection Authority and the National Bioethics committee (referral codes: VSN-15-241, VSN-09-098, and VSNb2015120006/03.01 with amendments, and VSN-16-093).

#### UKB ICV

The ICV processed data of 39 283 UK Biobank (UKB) participants subset of the 500 000 UKB study participants, was received for those who underwent an MRI acquisition.^29^ After the quality control checks, outliers’ removal, European ancestry filtering, and additional filtering a final set of 37 100, subjects were retained for the final study. The ICV phenotype (volume of estimated total intra cranial, whole brain) was retrieved from UKB using field code ‘26 521’ as described in Jansen *et al*.^10^ After the quality control criteria, the raw data were rank-transformed inverse normalized, and adjusted for known confounders (height, sex, age, age2, sex × age and pc1-pc20). The residual of the inverse normalized adjusted data was used as a quantitative variable for association testing. This study was approved through UKB license number 24 898.

#### ENIGMA ICV + EGG HC (head circumference)

The GWAS meta-analysis of ENIGMA ICV + EGG HC published by Haworth *et al*.^3^ was accessed through web-portal (link in URLs) and subsequently meta-analysed together with ICV data from Iceland and UKB.

### Genotyping and imputation

In the Icelandic data set, those with ICV measurements and with others phenotypes/traits participated in the number of projects at deCODE genetics. The preparation, chip-genotyping and or whole-genome sequencing of these samples was carried out at deCODE genetics.^30,31^ Using Graphtyper^32^ on WGS (using GAIIx, HiSeq, HiSeqX and NovaSeq Illumina technology to a mean depth of at least ×17.8), data of 61 205 Icelanders, 42.9 million high quality sequence variants were identified. Along with WGS set, deCODE genetics has also chip-genotyped 155 250 Icelanders using one of the Illumina genotyping arrays. The genotype calls (including SNP and insertions/deletions) based on WGS set were imputed into chip-genotyped subjects and long-range phased by using haplotype sharing and genealogical information.^33^

In the UKB, the samples were genotyped on two Affymetrix arrays. Initial set of 50 000 samples was chip-genotyped using the Affymetrix UK BiLEVE Axiom array.^34^ The additional set of 450 000 samples were chip-genotyped for 850 000 sequence variants using the Affymetrix UKB Axiom® array.^34^ Both arrays target 95% of same variants.^34^ The chip-genotypes were used to impute for additional markers by using 1000 Genomes phase 3,^35^ UK10K^36^ and HRC reference panels.

### Association analysis

We used linear mixed model implemented by BOLT-LMM^37^ on the normalized and adjusted ICV data assuming additive genetic model on Icelandic, and the UKB data set to test for association of 42.9 million sequence variants. For the quantitative traits, we assume that they follow a normal distribution. The LD score regression was used to account for inflation in the test statistics which may arise due to cryptic relatedness and population stratification.^38^ To compute the *P*-value, we used likelihood-ratio test applied through in-house software.^30^

### Meta-analysis

We performed GWAS meta-analysis of ICV using three GWAS summary statistics; ICV + HC from Iceland (*N*_ICV + HC_ = 15,497, male = 7,271, female = 8759), ICV from the UKB (*N*_ICV_ = 37,100, male = 19,381, female = 17 779) and published GWAS summary data of ICV + HC from ENIGMA + EGG^3^ (*N*_ICV + HC_ = 26 577). Before performing meta-analysis, the variants in three data sets were mapped to NCBI hg38 position, later the variants for UKB and ENIGMA + EGG data set were matched to the variants in the Icelandic data set based on the allele variation. We included variants that were properly imputed in all data sets, and which have a minor allele frequency > 0.1% in more than one cohort.

In total, we tested up to 42.9 million sequence variants for association with ICV. The GWAS results from the three data sets (Iceland, the UKB, the ENIGMA + EGG (the early growth genetics consortium)) were combined using a fixed effect inverse variance mode (based on the effect estimates and standard error) allowing different allele frequencies (of genotypes) in each population, where each data set was allowed to have different allele frequency of the tested genotypes but assumed to have same effect in each population. Moreover, to control for a heterogenetic effect of the markers tested in the populations, we used a likelihood ratio test (Cochran’s *Q*) and so evaluated their test statistics. Total variation in the estimates, which is due to heterogeneity, was estimated using *I^2^*statistic.

We used weighted Bonferroni threshold to find lead associations. To claim a novel genome-wide association, the sequence variants used in the meta-analysis were split into five classes based on their genome annotation, and the weighted significance threshold for each class was used.

### Polygenic risk score calculation

Polygenic risk score (PRS) for ICV were constructed in UKB, and in Iceland using genome-wide association meta-analysis by excluding the target population to avoid any bias in PRS estimates i.e. the ICV meta-analysis of Iceland + ENIGMA + EGG was used for PRS calculation in UKB. To construct the PRS, a set of high quality and well imputed 610 000 SNPs, spanning the whole genome, were used as described earlier.^39^ These markers are also used for long-range-phasing analysis.

To construct the PRS, the LDpred was used to derive the allele-specific weights, per-locus, for each SNP from predictor GWAS. Using LDpred, we constructed the PRS for seven weight thresholds i.e. roughly corresponds to *P*-value threshold 1, 0.3, 0.1, 0.03, 0.01, 0.003 and 0.001. In Iceland, we constructed the PRS for 172 015 directly chip-typed and well imputed subjects. We used the ICV phenotype data of 15 497 Icelanders to estimate the phenotypic variance explained by the ICV-PRS and best weight was used to perform PRS phenoscan in Iceland. We tested 5215 binary traits and 6290 quantitative traits in Iceland. Likewise, in UKB, we constructed PRS for 487 410 participants from UKB. The variance explained for ICV by the PRS in UKB is reported in Supplementary Fig. 1 and Supplementary Table 7. We tested 7378 binary traits and 8227 quantitative traits in UKB to test whether ICV PRS predicts phenotypic variance of any tested trait. We used Bonferroni threshold (*P*_threshold_ < 0.05/27 110 = 3.2 × 10^−6^) to report significant associations.

### ICV versus sMRI

The ICV measure summarizes the total/global size of the human brain. The local brain regions, though adjusted for total ICV, may or may not grow proportionally to the ICV. To test whether the genetic variants associated with ICV impact global and local volume differently, we used the 64 ICV associated sequence variants to test for their effect on 115 local ICV regions, defined through FreeSurfer,^40,41^ using the Aseg and Desikan-Killiany-Tourville atlases to label cortical and sub-cortical brain regions.^42^ The Bonferroni threshold (*P* < 0.05/64/115 = 6.8 × 10^−6^) was used to find significant associations.

### Gene expression (eQTL analysis) study using deCODE and GTEx data sets

We assessed *cis*-eQTL effects of the variants associated with ICV. For the GTEx data of 49 tissues, we retrieved precomputed eQTL estimates for all the genes (GTEx portal https://www.gtexportal.org/). Additionally, we used RNA sequence data of whole blood samples (*n* = 13 173) from deCODE Genetics.^43^ For both data sets, we included genes expressed more than one transcript per million (median value) that were defined in GENCODE 26 (GRCH38) within 1Mb of the ICV variants. Altogether, we tested 3310 moderately (median transcript per million > 1) or highly (median transcript per million > 10) expressed genes in 50 tissues and performed 75 728 (combination of variant × gene × tissue). Detailed description of eQTL analyses for deCODE data set is described here.^43^ The associations were significant if ICV variants are in high LD (*r*^2^ > 0.8) with top-eQTLs (*P*_threshold_ < 0.05/75 728 = 6.6 × 10^−7^).

### Proteomics data analysis

The proteomics data analysis (pQTL study) was performed using SOMAscan proteomics assays (SomaLogic, Inc.) of 4719 protein levels in plasma using proteomics and genotypes data of 35 559 Icelanders as described previously.^44^ For that we used pre-computed associations for *cis*/*trans*-pQTL signals. For 64 ICV variants, we tested whether ICV variants are in high LD (*r*^2^ > 0.8) with top *cis*/*trans*-pQTLs (*P*_threshold_ < 0.05/4719/64 = 1.7 × 10^−7^).

### Phenome-wide association scan using GWAS catalogue

We looked up ICV variants using NHGRI GWAS Catalogue^45^ (https://www.ebi.ac.uk/gwas/), updated on 8th June 2021, data of GWAS signals for 5004 traits. For phenome-wide association scan (pheWAS), we tested whether ICV variants are in high LD (*r*^2^ > 0.8) with reported GWAS associations.

### Genetic correlation analysis

To perform bivariate phenome-wide genetic correlation analysis between ICV meta-analysis and published studies, we used GWAS summary statistics data of 1483 traits^9,12,10,46–60^ (*P* < 0.05/1483 = 3.4 × 10^−5^). These studies are largely represented by Caucasian populations from different data-source each with effective sample size above 5000. To estimate the genetic correlations, we used LDSC^61,62^ where 1000 genome reference panel was used to estimate the LD structure. Major-histocompatibility complex region (6p22.1–6p21.3 which is about 30Mb size) was excluded due to its complex LD pattern. It is likely that most of the published GWAS traits contains a sub-sample from the UKB or other cohort that may/may not represent sample overlap; therefore, to eliminate the possibility that correlation estimates are inflated, we also performed genetic correlation of ICV meta-analysis using leave-one-sample out (ICV excluding UKB, or Iceland, or ENIGMA + EGG).

### Bidirectional causal analysis

MR analysis was used to estimate the causal effect of exposure on outcome trait. The GWAS significant variants robustly and independently associated with exposure trait were used as instrumental variables. The effect estimates from exposure were tested for causal effect on outcome trait. We used inverse-variance-weighted (IVW) regression, as well as Egger regression methods (implemented in MendelianRandomization package^63^) to estimate the causal effect. To compute *P*-value, we used *t*-distribution ‘*t*-dist’, and for standard error the ‘random model’ was used as implemented in MendelianRandomization package.^63^ Further to test whether the effect estimates by IVW are biased, we used MR Egger method that includes intercept. The Egger method specifically test for pleiotropy (i.e. intercept is different from zero) in IVW estimates.

### Data availability

The GWAS summary statistics for ICV meta-analysis will be made available at https://www.decode.com/summarydata/. Other data generated or analysed in this study are included in the article and its supplementary files. Additional GWAS data which was used to combine GWAS studies for meta-analysis can be accessed from respective resource as:

**ENIGMA + EGG meta-analysis summary statistics:** https://archive.mpi.nl/mpi/islandora/object/mpi%3A1839_ee7452e7_34c0_4d48_9598_5c14c0f89aae.

**ENIGMA + EGG GWAS data:** https://archive.mpi.nl/mpi/islandora/object/mpi:1839_3ab10f18_07d4_4171_8136_d0e0d100289f?asOfDateTime = 2018-11-12T10:36:05.617Z.

**Software/code availability (URLs):** We used the following publicly available software to analyse the data:

**GraphTyper** (v2.0-beta, GNU GPLv3 license) at https://github.com/DecodeGenetics/graphtyper.

**Svimmer** (v0.1, GNU GPLv3 license), the structural variant merging software at https://github.com/DecodeGenetics/svimmer.

**GCTA** (v1.93.3beta2) at https://yanglab.westlake.edu.cn/software/gcta/#Overview.

**LDPred** (v1.0.8) at https://github.com/bvilhjal/ldpred.

**LD score regression** at https://github.com/bulik/ldsc.

**qqman** package at https://github.com/stephenturner/qqman.

**MandelianRandomization** package at https://github.com/cran/MendelianRandomization.

**The genotype-tissue expression:** https://www.gtexportal.org/.

**NHGRI GWAS Catalogue:** ^45^ https://www.ebi.ac.uk/gwas/.

We used version 3.6.3 of R and version 1.2.5042 of RStudio for statistical analyses and for generating graphs and figures.

## Results

### GWAS meta-analysis for ICV

We meta-analysed GWASs of ICV and HC from Iceland (*n* = 15 497), ICV from the UKB (*n* = 37 100), and ICV and HC from enhancing neuroimaging genetics through meta-analysis (ENIGMA) consortium and Early Growth Genetics (EGG) consortium^3^ (*n* = 26 577) to search for sequence variants associated with ICV (Fig. 1). The meta-analysis included 42.9 million imputed variants available in the Icelandic^32^ and UK samples and 9.7 million in the ENIGMA/EGG sample.^3^ We applied a weighted Bonferroni approach that uses significance thresholds for variants based on their functional annotation class.^64^ The ICV meta-analysis yielded 57 variants that met the threshold for genome-wide significance, of which 30 represent novel ICV associations (Fig. 2, Supplementary Table 1 and Supplementary Figs. 2–31).

**Figure 1:**
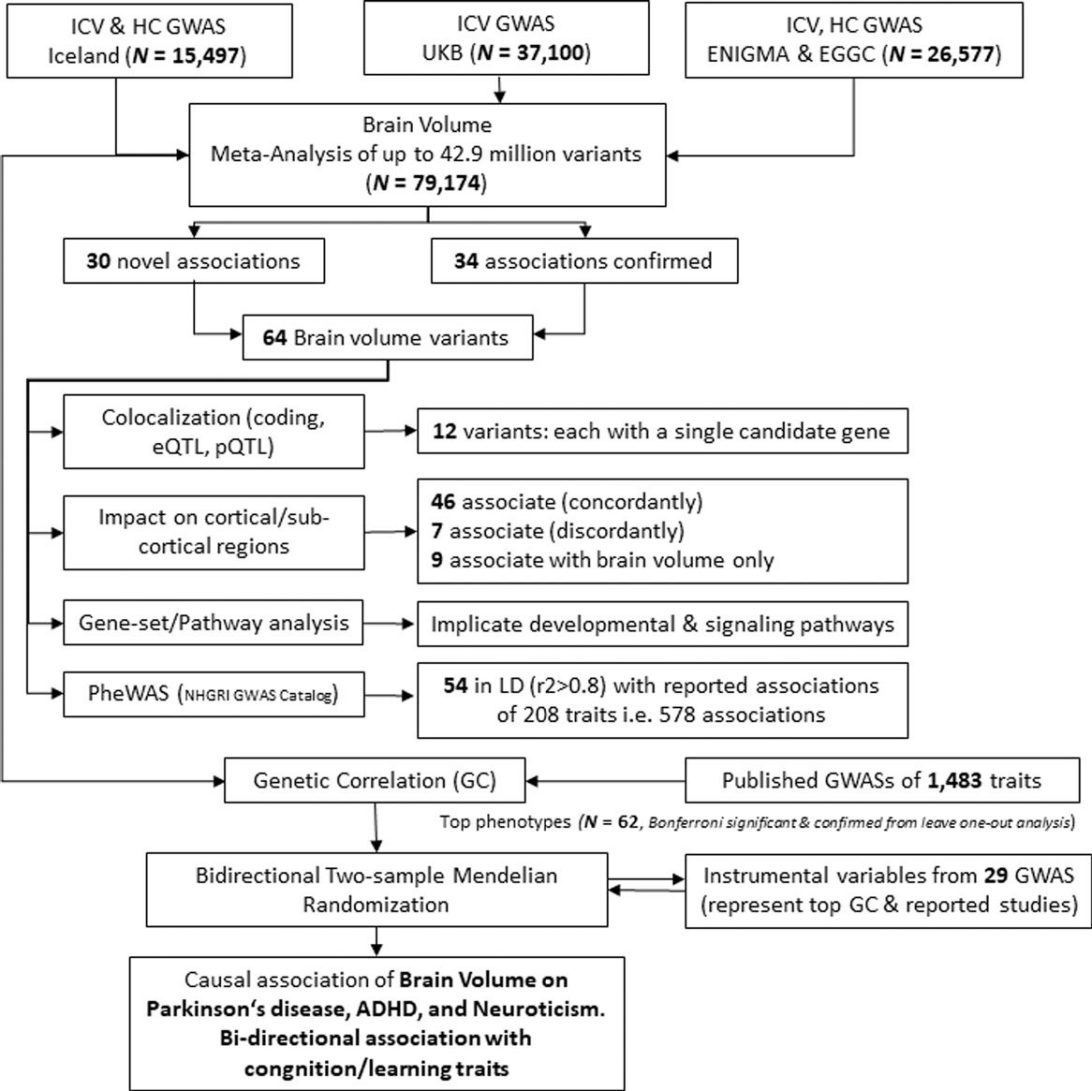
**Workflow of the study**. A GWAS meta-analysis of ICV by combining GWAS summary data from Iceland, UKB and ENIGMA + EGG (total *n* = 79 174) was performed. Our analysis identified 30 novel associations and confirmed 34 associations with ICV. For these 64 ICV associated variants, we performed *cis*-colocalization studies, studied their impact on cortical and sub-cortical regions (volumes), performed a PheWAS by looking up the variants and correlated variants up in the GWAS catalogue. Additionally, we studied the involvement of ICV associated genes in known pathways and gene-set terms. Finally, to understand the causal path of wide range of diseases, we used ICV associated variants (as instrumental variables) to study their impact on genetically correlated traits for Mendelian randomization analysis.

**Figure 2:**
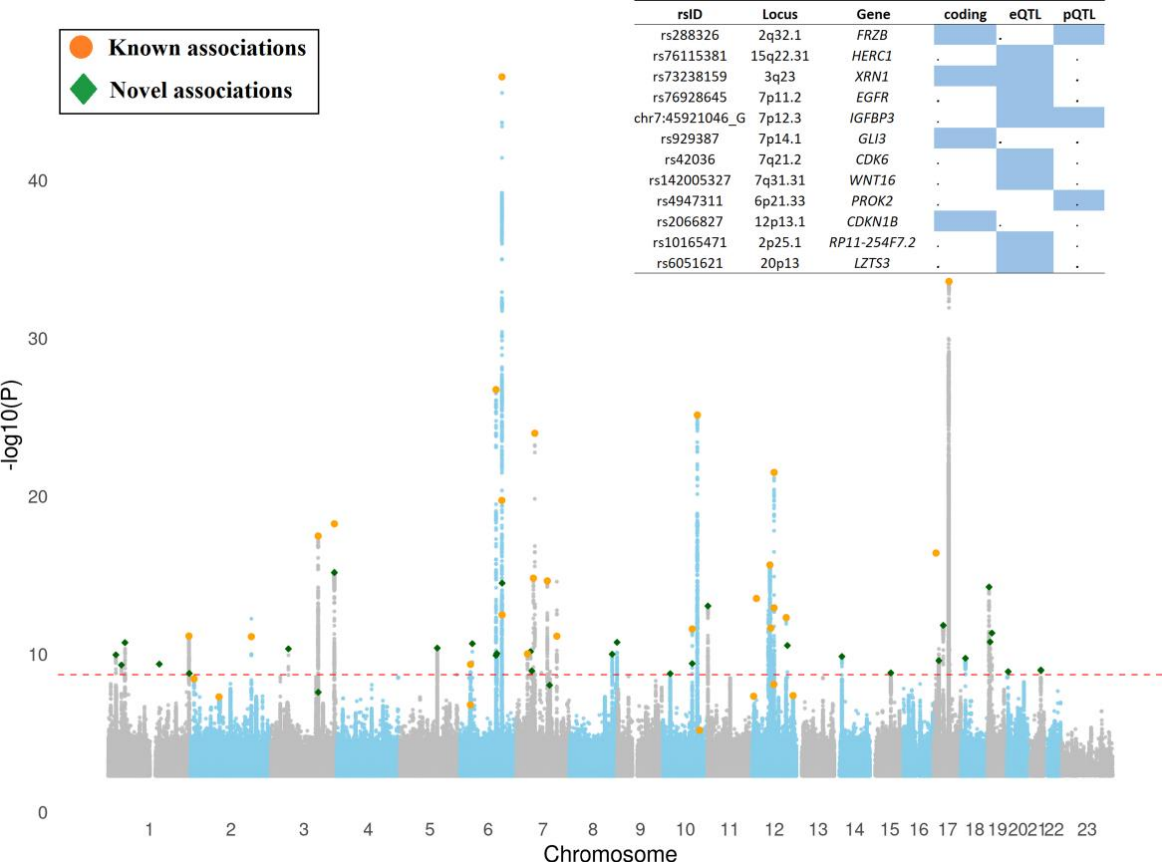
**Manhattan-plot showing association results for ICV (*n* = 79 174) with 42.91 million sequence variants (SNPs, In-dels and SVs)**. Each dot represents the position of a tested marker for the association. The *x*-axis represents the chromosomal position of the tested marker (where 23 refers to chromosome X), and the *y*-axis represents the significance (−log_10_*P*) of the observed association. The horizontal dotted line represents *P* = 1.2 × 10^−9^ (0.05/42.9 × 10^6^). Novel associations are highlighted with diamond shape, whereas bold filled dots represent associations of variants already reported in the scientific literature (Supplementary Table 1). The basic GWAS descriptive statistics calculated for ICV meta-analysis through LDSC^61,62^ are *h*^2^ = 0.0626 (0.0044 SE), lambda GC = 1.2818, mean Chi^2^ = 1.4252 and intercept = 1.0397 (0.0136 SE).

Previous GWAS studies on ICV or HC have reported 36 variants^10,3,11,65–67^ (Supplementary Table 1). Of those 36 variants, we replicated the association of 34 variants (*P*_threshold_ < 0.05, Supplementary Table 1). Altogether, 64 ICV associations at 51 loci, including 30 novel, were significant and explain 5.0% of the variability in ICV (Supplementary Table 1 and Supplementary Fig. 32 compares effect sizes between the studies**)**. The largest effect on ICV was conferred by a low-frequency variant at 6p21.2 (rs180819997-A, β = −0.191 SD, *P* = 2.2 × 10^−11^, EAF = 1.05%, Supplementary Table 1). This variant has recently been shown to associate with height^68^ (*P* = 4.9 × 10^−9^). However, the effect sizes were not reported,^68^ and we tested the association with height using UKB + Icelandic data (β = −0.07, *P* = 3.3 × 10^−9^*, n* = 511 260). The effect estimates (in standard units) of rs180819997-A on ICV (adjusted for height) is larger than that on height (*P*_heterogeneity_ = 9.0 × 10^−5^).

### Identification of candidate genes

To search for genes mediating the effects at the ICV loci, we determined whether the lead ICV variants or correlated ones (*r*^2^ > 0.8) affected amino-acid sequence (coding), mRNA expression (*cis*-eQTL) and/or protein levels in plasma (*cis*/*trans*-pQTL). Ten of the 64 ICV variants are in high LD (*r*^2^ > 0.8) with coding variants (missense or loss-of-function) in *MYCL*, *CDKN1B*, *HOOK2*, *FRZB*, *TGOLN2*, *XRN1*, *TNNC1*, *GLI3*, *ZNF789* and *LRRC24* (Supplementary Table 1).

We performed colocalization analysis of the 64 ICV variants with variants affecting transcript abundance using sequencing of RNA from whole blood samples (*n* = 13 173) as well as 49 tissues from GTEx (v8).^69^ Altogether, we tested 3310 moderately/highly expressed genes present within 1 Mb of the 64 variants in 50 tissues; performed 75 728 independent tests (combination of variant × gene × tissue tested, *P*_threshold_ < 0.05/75 728 = 6.6 × 10^−7^). Twenty-six ICV variants colocalize (or are in high LD *r*^2^ > 0.8) with the top *cis*-eQTLs in any of the tissues analysed, regulating the expression of 71 genes (Supplementary Table 2A).

We also performed pQTL analysis using SOMAscan proteomics assays (SomaLogic, Inc.), measuring 4907 aptamers targeting 4719 proteins in 35 559 Icelanders to test for association between ICV variants and protein levels in plasma.^70^ Five of the 64 ICV variants are in high LD (*r*^2^ > 0.8) with top pQTL (*P*_threshold_ < 0.05/4907/64 = 1.6 × 10^−7^) (Supplementary Table 2B). Of these five, two are *cis*-pQTL (*FRZB and IGFBP3*) and three are *trans*-pQTLs (*HS6ST2*, *PROK2* and *CR2*).

We subsequently integrated the results of these three analyses (*cis*-coding, *cis*-eQTL and *cis*/*trans*-pQTL) to search for genes mediating the effect of variants on ICV. In the case of 12 ICV variants, our analysis implicated a single protein coding gene (*CDKN1B*, *GLI3*, *FRZB*, *LZTS3*, *XRN1*, *WNT16*, *HERC1*, *IGFBP3*, *EGFR*, *CDK6 and PROK2*) and one long non-coding RNA (*RP11-254F7.2*) (Supplementary Table 1).

### Phenome-wide association study (PheWAS)

We determined whether the 64 ICV variants colocalize (*r*^2^ > 0.8) with variants reported to associate with 5004 traits in the NHGRI GWAS Catalogue^45^ (URL). Of the 64 ICV variants, 54 colocalize with 578 reported associations other than ICV (Supplementary Table 3) of which, 20 colocalize with various blood trait variants, 17 with anthropomorphic measurement variants (height, BMI and waist circumference), 14 with brain region variants (volume, area and thickness), 12 with personality traits and cognitive measures, 11 with cardiovascular disorder variants and the rest colocalized with variants associated with neurological disorders, autoimmune disorders and reproductive traits (Supplementary Table 3).

### Impact on cortical and sub-cortical regions

ICV correlates phenotypically (Supplementary Table 4A), and genetically^8^ with the volumes of various cortical and sub-cortical regions. We tested the 64 ICV variants for their association with 115 cortical and sub-cortical volumes (adjusted for ICV) extracted from structural brain MRI (sMRI) data of 37 100 participants from the UKB. Of the 64 ICV variants, 53 associate with at least one sMRI trait (*P*_threshold_ < 0.05/64/115 = 6.8 × 10^−6^) (Supplementary Table 4B).

### Gene set enrichment and pathway analysis

To understand the molecular mechanism and impact of ICV variants on known pathways/gene-sets, we performed gene enrichment/pathway analysis using the molecular signature database^71^ through MAGMA.^72^ MAGMA uses full GWAS summary statistics to test for regional association of genes and then uses all genes as an input for the pathway and enrichment analysis by adjusting for known confounders.^72^ The analysis found enrichment of 90 pathways/gene set terms (*P*_threshold_ < 0.05/9753 = 5.1 × 10^−6^) including terms describing insulin signalling, brain ventricle development, oncogenesis, neurogenesis, growth/development of cells, metabolic processes, and facial and skull development (Supplementary Table 5).

### Genetic correlation analysis

**Figure 3:**
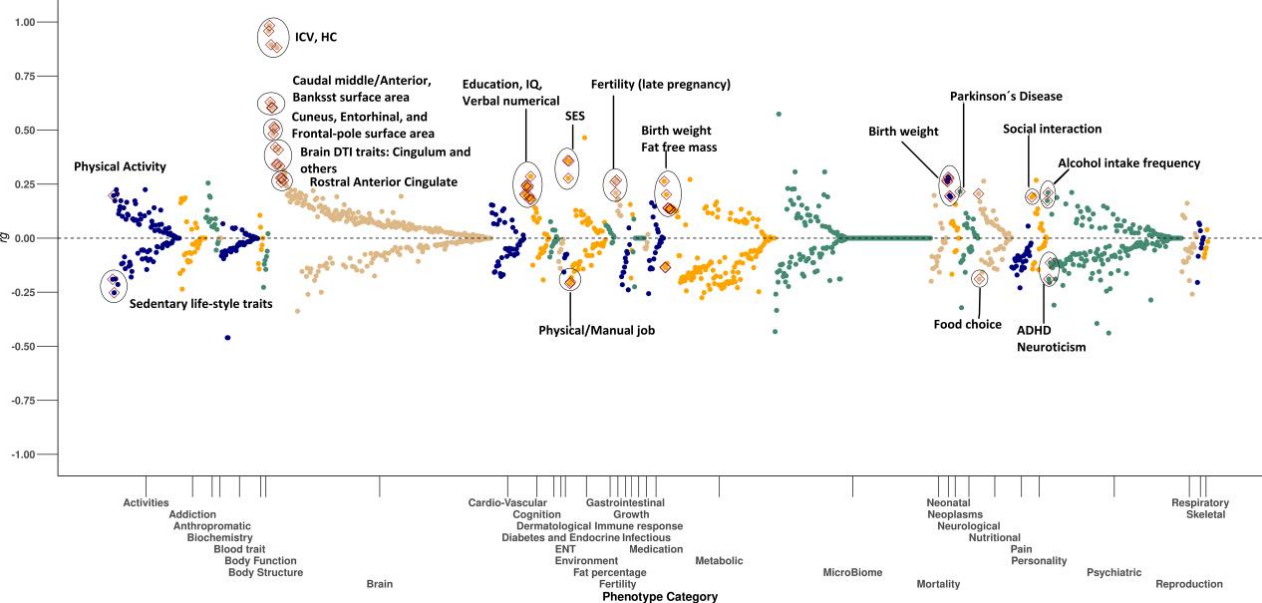
**Phenome-wide bivariate genetic correlation between ICV and 1483 published GWAS studies estimated through LDSC**.^61,62^ Each dot is an estimate of genetic correlation (*rg*) between ICV GWAS meta-analysis and one of the tested GWAS traits (binned into phenotype categories), where the *x*-axis represents phenotype (category) and the *y*-axis shows its genetic correlation (*rg*). The significant associations (*P*_threshold_ < 0.05/1483 = 3.37 × 10^−5^) are highlighted with diamond shape.

We used LD score regression to perform a phenome-wide genetic correlation analysis between the ICV meta-analysis and 1483 published GWAS studies (Supplementary Table 6A).^61,62^ These studies include GWAS data of brain anatomy and physiology, neurological, metabolic, anthropometric, cardiovascular and blood traits. Our analysis highlighted 62 of the 1483 GWAS traits showing genetic correlation with ICV meta-analysis after correcting for multiple testing and leave-one-sample-out analysis (*P*_threshold_ < 0.05/1483 = 3.4 × 10^−5^, Fig. 3, Supplementary Table 6A). Among the positive genetic correlations, we replicated genetic correlation of GWASs of the brain’s cortical and sub-cortical regional volumes,^12^ educational attainment,^10^ cognitive performance^10^ (verbal and numerical reasoning) and Parkinson’s disease.^9^ Furthermore, ICV exhibited positive genetic correlation with social interaction, neonatal traits, nutritional choice and higher alcohol intake. We also replicated the previously reported negative genetic correlation of ICV with ADHD and neuroticism.^8^ Moreover, ICV correlated negatively with having a physical job, loneliness and sedentary lifestyle (Supplementary Table 6A). Some of these genetic correlations are expected, such as other brain anatomy phenotypes. We further investigate ADHD and Parkinson’s disease, since these are the only disease/disorder phenotypes that survive our multiple testing threshold. We also focus on cognition and learning phenotypes, since we have access to large cohorts in Iceland and UKB for further analysis.

### Phenotypic correlations

We tested the phenotypic correlations between ICV and selected traits: ADHD, Parkinson’s, neuroticism and cognitive/learning traits in Icelandic and UKB data. The UKB Parkinson’s disease cases have larger ICV (cases = 83, controls = 37 154, β = 0.30 SD, *P* = 0.0072), which is consistent with a previous report,^73^ and in Iceland, we find that ADHD cases have smaller HC (cases = 5489, controls = 45 291, β = −0.15, *P* = 1.3 × 10^−26^). The phenotypic correlations for the tested traits are in keeping with the genetic correlation results (Supplementary Table 6B).

### Polygenic risk score analysis

We constructed two PRSs for ICV, one excluding UKB (ICV-PRS-exUKB) and the other excluding Iceland (ICV-PRS-exICE), to allow estimation of phenotypic variance explained in the UKB and Icelandic samples, respectively. ICV-PRS-exUKB explained 5.9% of phenotypic variance in UKB (*N*_ICV_ = 37 100), and ICV-PRS-exICE explained 8.8% of phenotypic variance in Iceland (*N*_ICV_ = 1328) (Supplementary Fig. 1 and Supplementary Table 7).

### Mendelian randomization analysis

A recent study examined the shared heritability of common neurological disorders using genetic correlation data.^74^ We employed a two-sample MR approach using the 64 ICV associated variants and top variants from available meta-analyzes as instrumental variables (IVs) to study the causal effect on traits genetically correlated with ICV as well as the traits studied by Anttila *et al*.^74^ (There are large meta-analyses available in several categories: neurological diseases/disorders, personality and behavioural traits, and cognition/learning/neonatal traits)^9,48–60,75–77^ (Supplementary Table 8A, *P*_threshold_ < 0.05/35 = 1.4 × 10^−3^).

**Figure 4:**
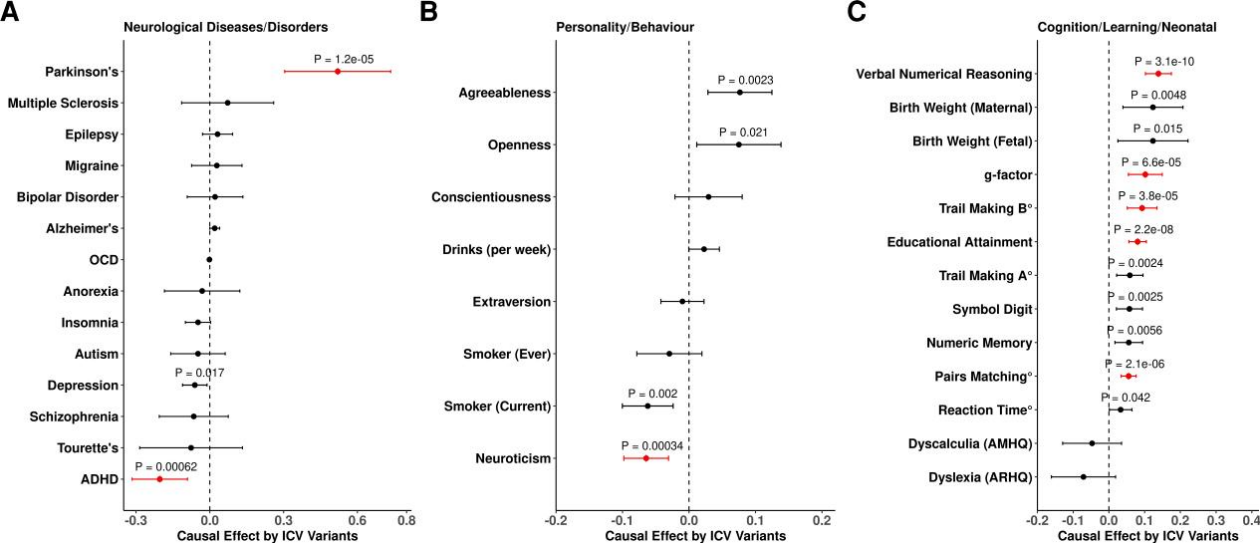
**Causal association of instrumental variants from ICV on 34 tested traits**. (**A**) neurological diseases/disorders, (**B**) personality/behaviour traits, (**C**) cognitive/learning/birth weight traits. The analysis was performed using a two-sample Mendelian randomization (MR) approach, the instrumental variables and their effect sizes are based on results for ICV variants versus their effects from largest available studies of the genetically correlated traits (Supplementary Table 8A). IVW (inverse variance weighted) method was used to estimate the causal effect, additionally Egger analysis was performed to detect whether IVW estimates are biased i.e. intercept is different from zero (Supplementary Table 8A). The Bonferroni significant associations (*P* < 0.05/34 = 1.47 × 10^−3^) are highlighted, ‘°’ refers to traits for which effect estimates were flipped for better representation (Supplementary Figs. 33–46, Supplementary Table 8A and 8B).

**Figure 5:**
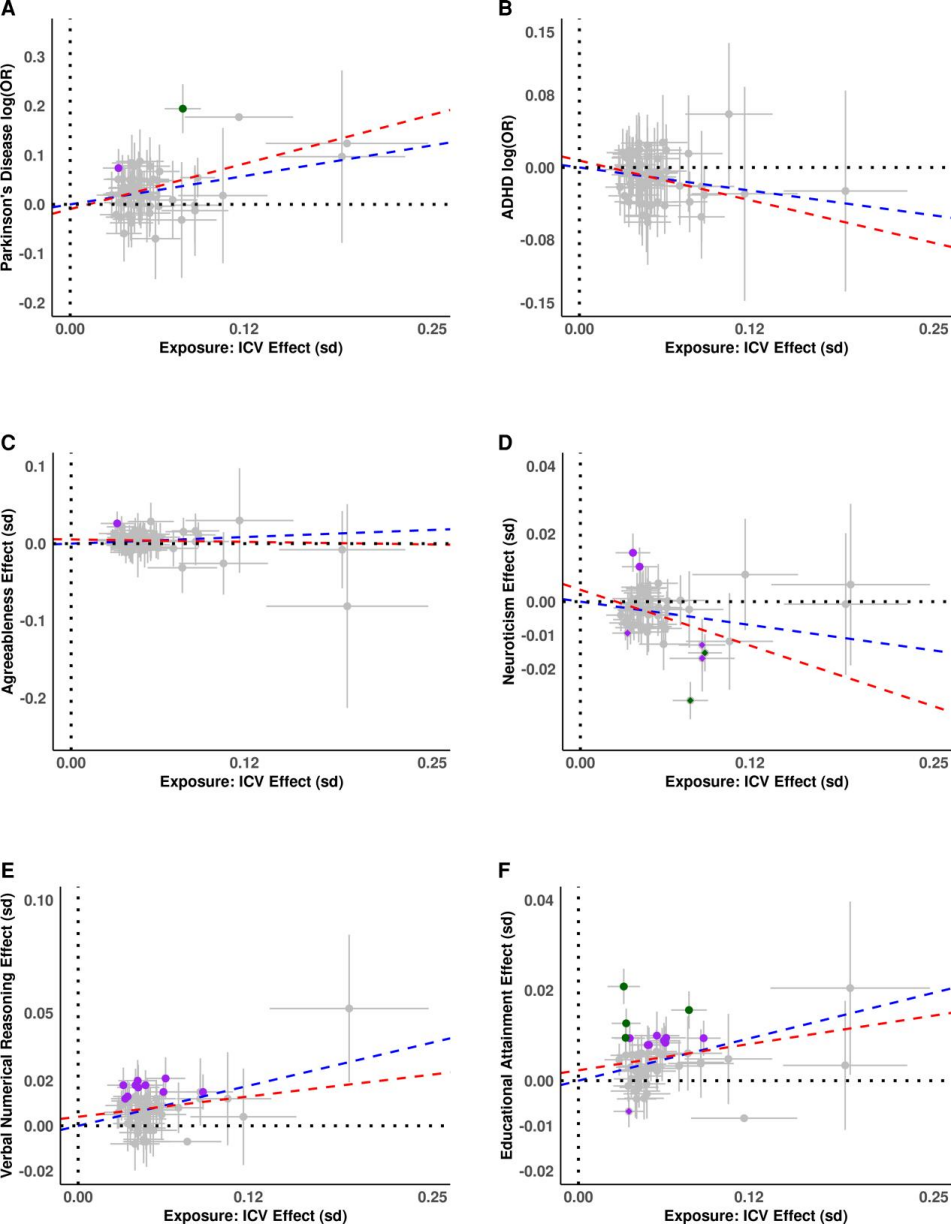
**Effect versus effect plots of top associations from MR analysis**. On *x-*axis are effect size for ICV and on y-axis (not always symmetric around zero) for; (**A**) Parkinson’s disease as log(odds ratio), (**B**) ADHD as log(odds ratio), (**C**) Agreeableness as beta in SD, (**D**) neuroticism as beta in SD, (**E**) verbal numerical reasoning as beta in SD and (**F**) educational attainment as beta in SD. All effects are plotted for alleles with increasing ICV. Blue line represents the estimated slope from IVW (inverse variance weighted regression), and red line is estimated from MR Egger analysis including the intercept. Green dots represent conventional GWAS associations (*P* < 5.0 × 10^−8^) for respective *y*-axis trait, while purple dots are Bonferroni significant associations (*P* < 0.05/64 = 7.8 × 10^−4^) for respective the *y*-axis trait. See Supplementary Figs. 33–46 for individual trait plots.

The MR results were consistent with positive causal effects of ICV variants on Parkinson’s disease (β = 0.520, *P* = 1.22 × 10^−5^), and cognitive traits; including verbal numerical reasoning/fluid intelligence (β = 0.139, *P* = 3.07 × 10^−10^), *g* factor (β = 0.102, *P* = 6.62 × 10^−5^), trail making test B (β = −0.093, *P* = 3.79 × 10^−5^), educational attainment (β = 0.073, *P* = 9.18 × 10^−8^), and pairs matching (β = −0.055, *P* = 2.11 × 10^−6^) and a negative causal effect on ADHD (β = −0.203, *P* = 6.16 × 10^−4^), and on neuroticism (β = −0.064, *P* = 3.37 × 10^−4^) (Fig. 4, Supplementary Table 8B). For Parkinson’s disease, this corresponds to a 68% increase in disease risk per SD of ICV and an 18% decrease in the risk of ADHD per SD of ICV. The effect estimates from the 35 tested outcome studies are mostly made up of samples of Caucasian origin and may contain some overlap of samples from the UKB or ENIGMA or EGG used in our ICV meta-analysis. To evaluate whether these results are driven by sample overlap, we performed MR analyses using ICV GWAS data with leave-one-sample-out (i.e. ICV meta-analysis excluding UKB, or Iceland, or ENIGMA + EGG GWAS (Supplementary Table 8A). All MR associations remained significant in the leave-one-sample out analyses (Supplementary Table 8B). Egger analysis of ICV variants with eight significant traits from inverse variance weighted (IVW) analyses revealed no evidence of variant pleiotropy, i.e. the intercepts were not significantly different from zero (Fig. 5, Supplementary Figs. 33–46 and Supplementary Table 8B).

To test for reverse causation, we used as IVs GWAS significant variants from Parkinson’s disease^9^ (*n* = 90), neuroticism^14^ (*n* = 135), insomnia^78^ (*n* = 780), ADHD^50^ (*n* = 9), depression^48^ (*n* = 98), schizophrenia^51^ (*n* = 113), bipolar disorder^52^ (*n* = 139), anorexia^53^ (*n* = 8), Alzheimer’s disease^75^ (*n* = 30), epilepsy^57^ (*n* = 10), multiple sclerosis^79^ (*n* = 275), smoking and alcohol drinking behaviour^58^ (*N*_smoking_ = 268; *N*_drinking_ = 72), migraine^80^ (*n* = 121), educational attainment^60^ (*n* = 1262), intelligence^60^ (*n* = 242) and birth weight^59^ (*n* = 144) as exposures for their potential causal effects on ICV (Supplementary Table 8C).

The exposures of Parkinson’s disease, neuroticism and migraine had a nominally significant effect on ICV in our MR analysis, but ADHD was not significant as an exposure for ICV (*P*_threshold_ < 0.05/29 = 1.7 × 10^−3^, β_ADHD_ = 0.019, *P*_ADHD_ =0.456; β_Parkinson’s_ = 0.040, *P*_Parkinson’s_ = 0.001; β_neuroticism_ = 0.126, *P*_neuroticism_ = 0.011; β_migraine_ = −0.042, *P*_migraine_ = 0.016; Supplementary Fig. 47 and Supplementary Table 8D). Effects of birth weight, insomnia, cognitive and learning trait IVs associated with ICV after accounting for multiple testing (*β*_birth-weight_ = 0.216, *P*_birth-weight_ = 1.16 × 10^−6^; β_insomnia_ = −0.192, *P*_insomnia_ = 7.07 × 10^−6^; β_education_ = 0.264, *P*_education_ = 4.11 × 10^−33^; β_cognitive performance_ = 0.190, *P*_cognitive performance_ = 8.62 × 10^−9^; Supplementary Table 8D).

## Discussion

Our GWAS meta-analysis of human ICV doubles the number of identified variants and provides novel insights into the biology of brain structure. Our analyses of transcriptome, proteome and coding variants highlight that 12 of the 64 ICV variants likely affect ICV via a single candidate gene each. Three of these genes (*GLI3, CDK6 and FRZB*) have a priori been associated with phenotypes closely aligned with ICV/skull size. Fifty-five variants are associated with various other traits, including personality/cognition/learning, cardiovascular disorders, neurological and autoimmune disorders. We also observe a general confluence of effects in analysis using multiple markers, such as genetic correlation and MR.

Three of the markers are related to genes previously associated with microcephaly or skull bone development, phenotypes closely related to ICV. One of these is a common missense variant in *GLI3* (p.Asp1137Asn) associating with larger ICV. Rare loss of function mutations in *GLI3* have previously been associated with a premature fusing of the skull (craniosynostosis).^81^ Therefore, we speculate that p.Asp1137Asn may associate with a delayed fusing of the skull. The second variant is common and associates with smaller ICV and further associates with lower expression of *CDK6*. This finding is consistent with a reported recessive association between a missense variant in *CDK6* and microcephaly.^82^ Thirdly, we find a common variant in *FRZB* (p.His488Gln) that associates with larger ICV and higher FRZB protein expression. *FRZB* plays a role in osteogenesis.^83,84^ The role of FRZB in osteogenesis suggests that *FRZB* may exert its impact on ICV by influencing skull development. Genome-wide significant associations with brain morphology have been reported in or near 5 of the 12 genes (*FRZB,*^85^*EGFR,*^86^*IGFBP3,*^87^*GLI3*^87^ and *CDK6*^88^). Other interesting associations reported in the GWAS catalogue include Parkinson’s disease (*LZB3*), educational attainment (*FRZB* and *GLI3*) and ADHD/Externalising behaviour (*HERC1*). Other, less related phenotypes were also associated directly with most of the markers. The pheWAS of the ICV variants reveals that 55 of the 64 variants associate with a wide range of diseases and traits, including personality/cognition/learning, cardiovascular disorders, neurological and autoimmune disorders. Particularly, one previously studied marker is the one that tags the inversion polymorphism located at 17q21.31. The inversion has two haplotypes in Caucasian populations, H1 and H2. H1 associates with Parkinson’s disease^9^ and larger ICV. One of the genes affected by the inversion polymorphism is *MAPT*, a candidate gene in Parkinson’s disease. H2, the inverted haplotype, associates with smaller ICV, neuroticism^14^ and negatively with cognitive traits.^60^ In order to understand the general confluence of the variants, we further analysed the data using genetic correlation and MR.

The phenome-wide genetic correlation analysis for ICV and 1483 published GWAS studies revealed genetic correlations with 62 traits. Of these 62 traits, we find two that are diseases or disorders, namely ADHD and Parkinson’s disease. One of those is related to neurodevelopment while the other is related to neurodegeneration. It is a key question whether variants associated with structural changes in the brain cause neurological disorders or alternatively whether genetic predisposition to certain neurological or neurodevelopmental disorders impacts brain structure or development. We attempted to dissect the causal relationships between ICV and genetically correlated traits using bi-directional MR analyses.

It is well established that ADHD correlates with smaller HC.^89^ However, few studies report a relationship between Parkinson’s disease and ICV.^73^ Here, we observe that Parkinson’s cases have greater ICV than controls. Our MR analysis is consistent with ICV associated variants having a causal effect on these diseases. A fundamental assumption of MR analysis is the absence of horizontal pleiotropy,^90^ i.e. the ICV sequence variants used as instruments should not systematically associate with another phenotype than ICV that causally affects the outcome. This assumption is inherently impossible to validate. However, because of the strength of the relationship between the effects of ICV variants and their disease effects, i.e. for each SD of ICV, the risk of Parkinson’s disease increases by 68% and the ADHD risk decreases by 18%, it is likely that alternative phenotypes driving this relationship would have to be strongly correlated with ICV. The reverse relationship between the effects of Parkinson’s disease and ADHD variants and their effect on ICV was weaker, suggesting that ICV, or its close correlates, are likely to drive or contribute more to the relationship rather than these disorders affecting ICV.

Nalls *et al*.^9^ have previously reported a significant causal effect of educational attainment on Parkinson’s disease via MR (effect = 0.162, SE = 0.040, *P* = 2.06 × 10^−4^). The reported causal effect of educational attainment with Parkinson’s disease is weaker than that of ICV with Parkinson’s disease (effect = 0.537, SE = 0.105, *P* = 4.74 × 10^−6^, Supplementary Table 8B). In comparison, the causal effect of ICV on educational attainment is small (effect = 0.08, SE = 0.012, *P* = 2.24 × 10^−8^, Supplementary Table 8B). The difference in significance is largely due to statistical power of these exposure phenotypes, where the educational attainment phenotype’s sample size is over a million, compared with 80K for ICV. With this evidence we conclude that ICV is a more probable explanation as an exposure conferring risk for Parkinson’s disease compared with educational attainment. This largest GWAS meta-analysis of ICV to date highlights 64 associations, of which 30 are novel. We implicate 12 genes through co-localization analyses. Our MR analyses revealed that ICV, or a closely correlated trait, has a causal effect on a neurodevelopmental disorder (ADHD) as well as on a neurodegenerative disease (Parkinson’s). These findings highlight the relationship between anatomical variation and neurological and developmental disorders, underscoring the potential for applying brain volume measures, combined with genetics, to gain a foothold in understanding the complex structure-function relationships of the brain.

## Supplementary Material

fcac271_Supplementary_Data

## Abbreviations

ADHD =: attention deficit hyperactivity disorder
BMI =: body mass index
CNV =: copy number variant
eQTL =: expression quantitative trait locus
GWAS =: genome-wide association study
HC =: head circumference
ICV =: intracranial volume
IV =: instrumental variables
IVW =: inverse-variance-weighted
LD =: linkage disequilibrium
MR =: Mendelian randomization
MRI =: magnetic resonance imaging
pQTL =: protein quantitative trait locus
SNP =: single nucleotide polymorphism
