# Supplementary material for: Thirty novel sequence variants impacting human intracranial volume
Source: Brain Commun. 2022 Oct 25;4(6):fcac271. doi: 10.1093/braincomms/fcac271 (PMC9677475; doi:10.1093/braincomms/fcac271)
Supplement: fcac271_Supplementary_Data [file fcac271_supplementary_data.zip › Supplementary_figures_and_locus_plots_Nawaz_MS_30082022.pdf]

Thirty novel sequence variants impacting human intracranial volume

Nawaz MS., et al.

Supplementary Figures

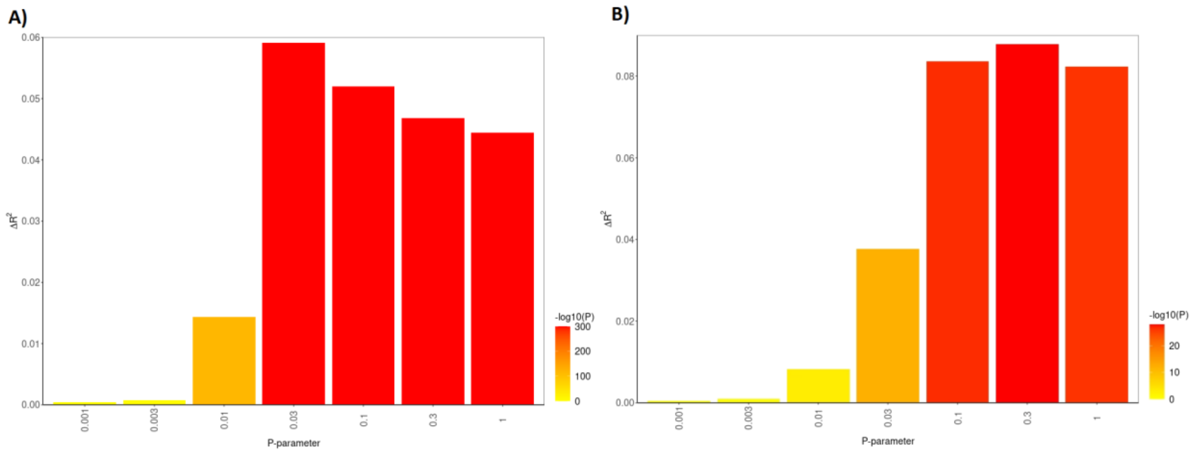

Supplementary figure 1: Phenotypic variance explained through intracranial volume polygenic risk score (PRS) (**A**) in UKB by intracranial volume PRS from Iceland+ENIGMA+EGGC (**B**) in Iceland by intracranial volume PRS from UKB+ENIGMA+EGGC. On x-axis are p-parameters (roghly corresponds to p-value threshold) used to construct PRS by LDSC, and on y-axis is $\Delta \boldsymbol{R}^2$ (variance explained) by PRS and color of bar corresponds to –log10(P) of association. Top associations: In UKB (A) p-parameter 0.03 explains 5.92% of phenotypic variance, and in Iceland (B) p-paramter ‚0.3‘ explains 8.78% of phenotypic variance.

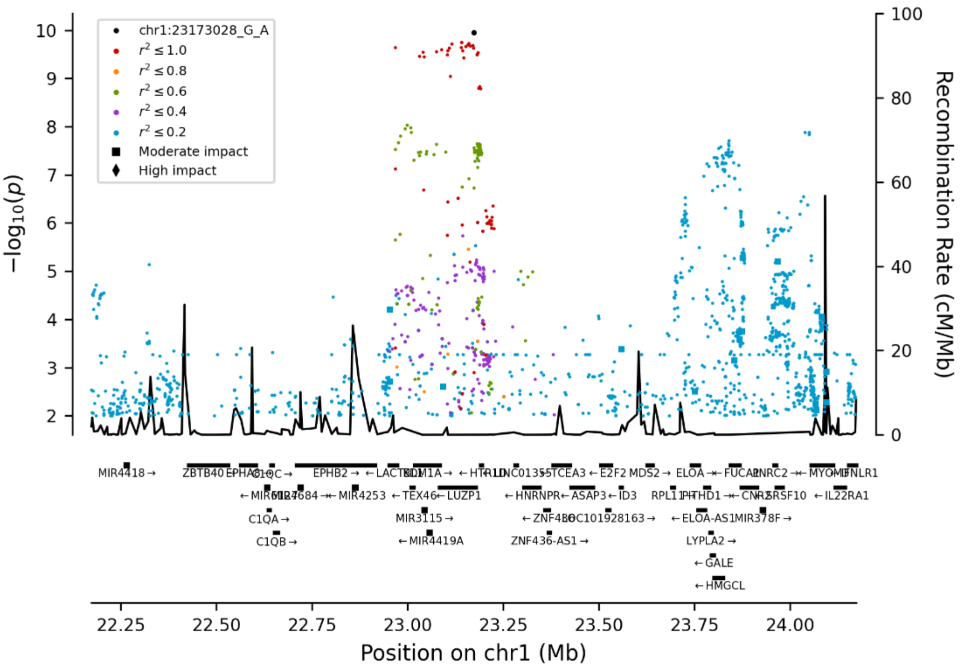

Supplementary locus plot 2: Locus plot of novel variant, rs148113724, with ± 500Kb flanking region, the color code represnt r^2 which is a measure (LD) of the lead variant with surrounding variants.

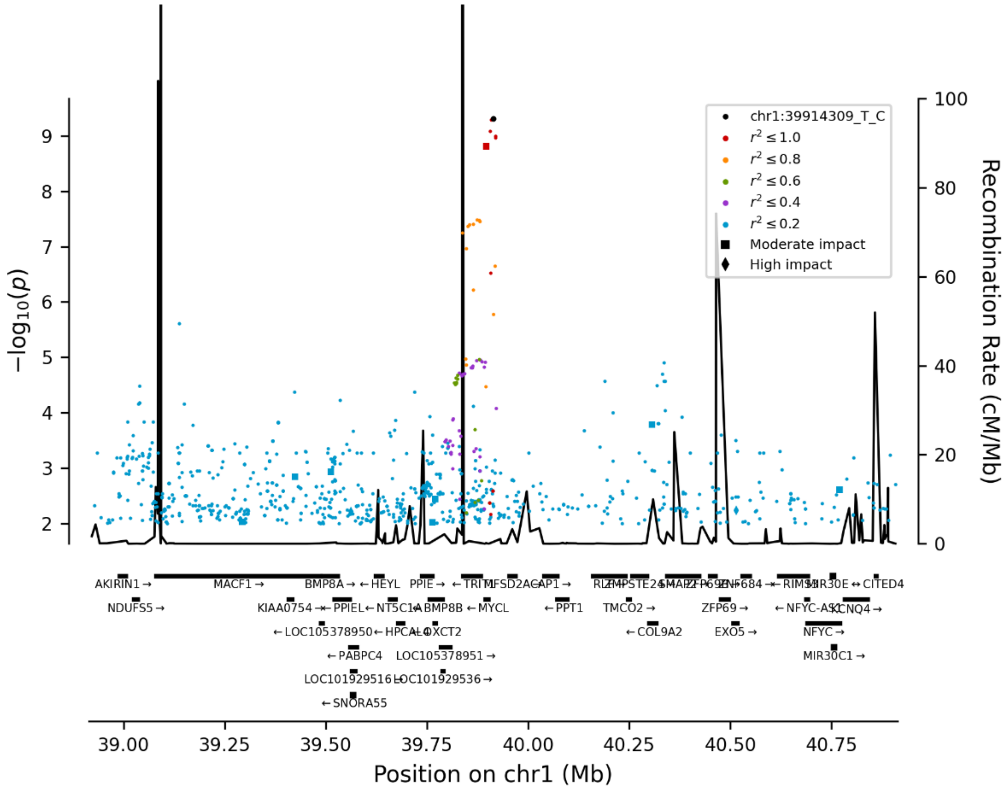

Supplementary locus plot 3: Locus plot of novel variant, rs12124681, with ± 500Kb flanking region, the color code represnt r^2 which is a measure (LD) of the lead variant with surrounding variants.

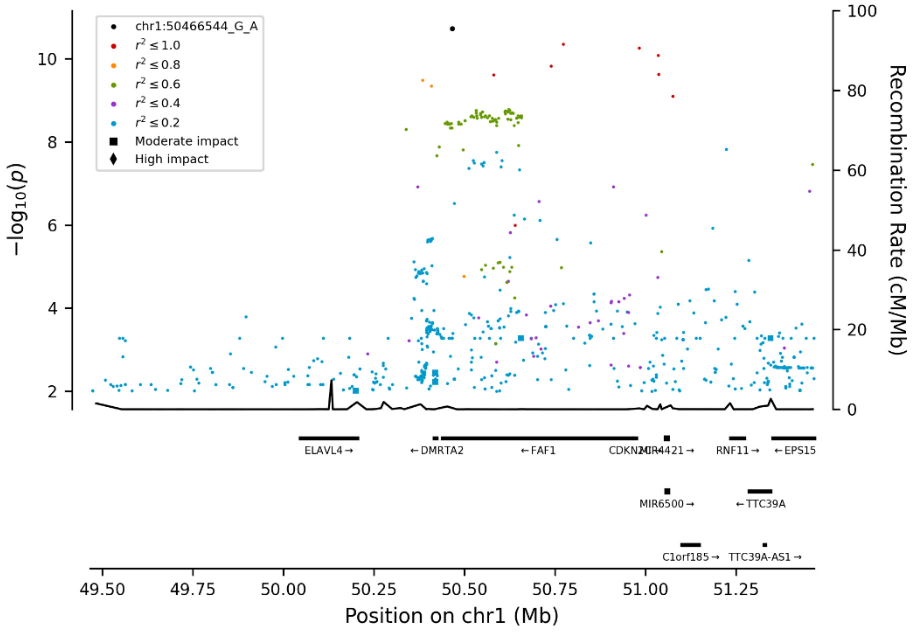

Supplementary locus plot 4: Locus plot of novel variant, rs115154906, with ± 500Kb flanking region, the color code represnt r[2] which is a measure (LD) of the lead variant with surrounding variants.

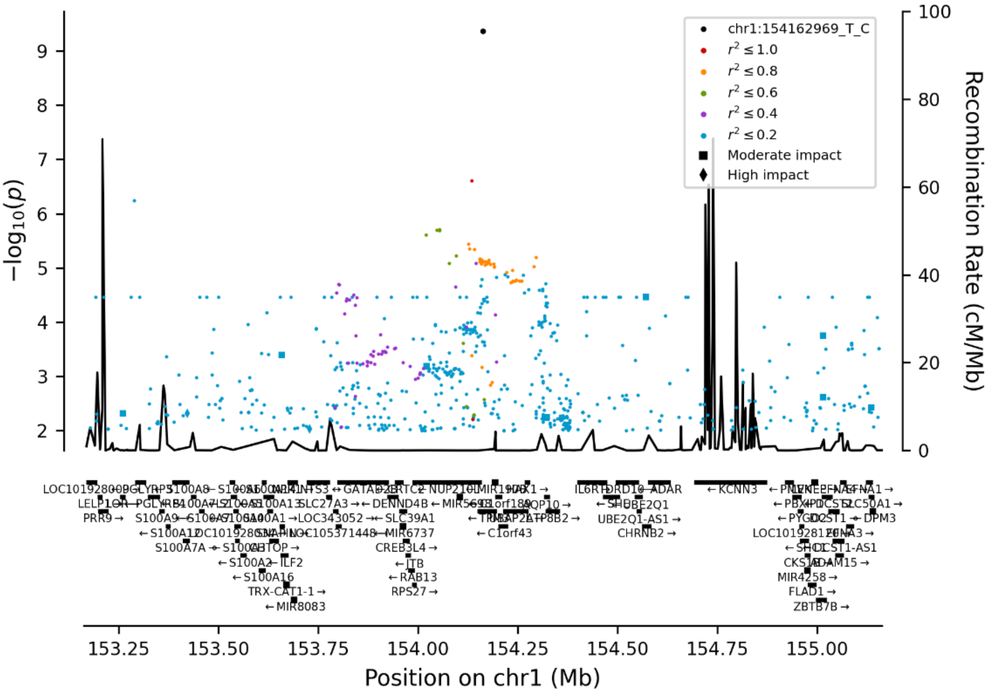

Supplementary locus plot 5: Locus plot of novel variant, rs4446955, with ± 500Kb flanking region, the color code represnt r[2] which is a measure (LD) of the lead variant with surrounding variants.

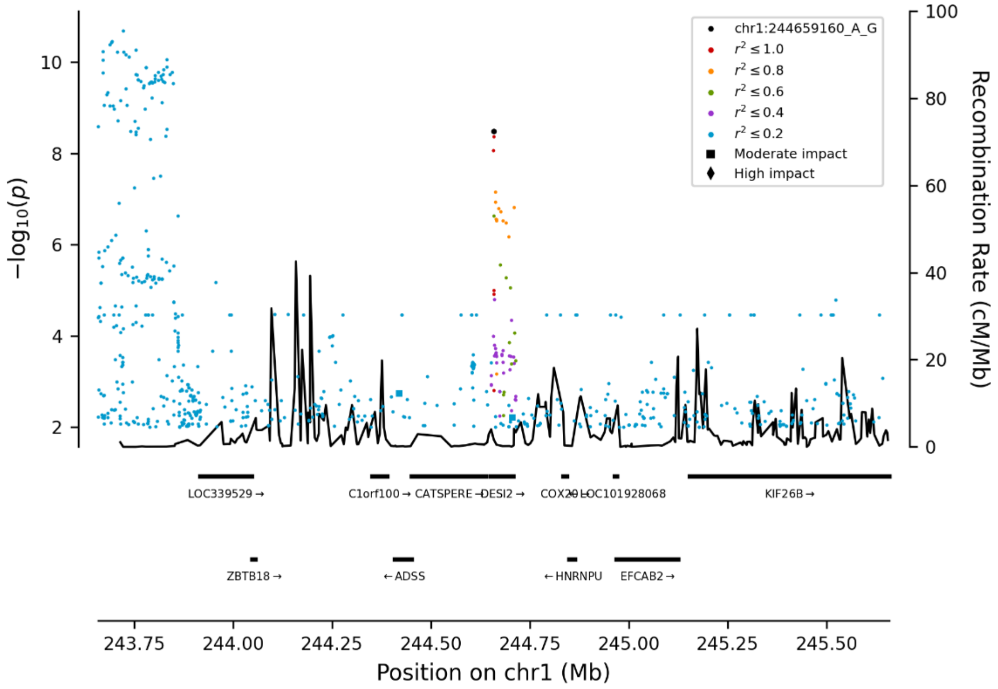

Supplementary locus plot 6: Locus plot of novel variant, rs10927290, with ± 500Kb flanking region, the color code represnt r^2 which is a measure (LD) of the lead variant with surrounding variants.

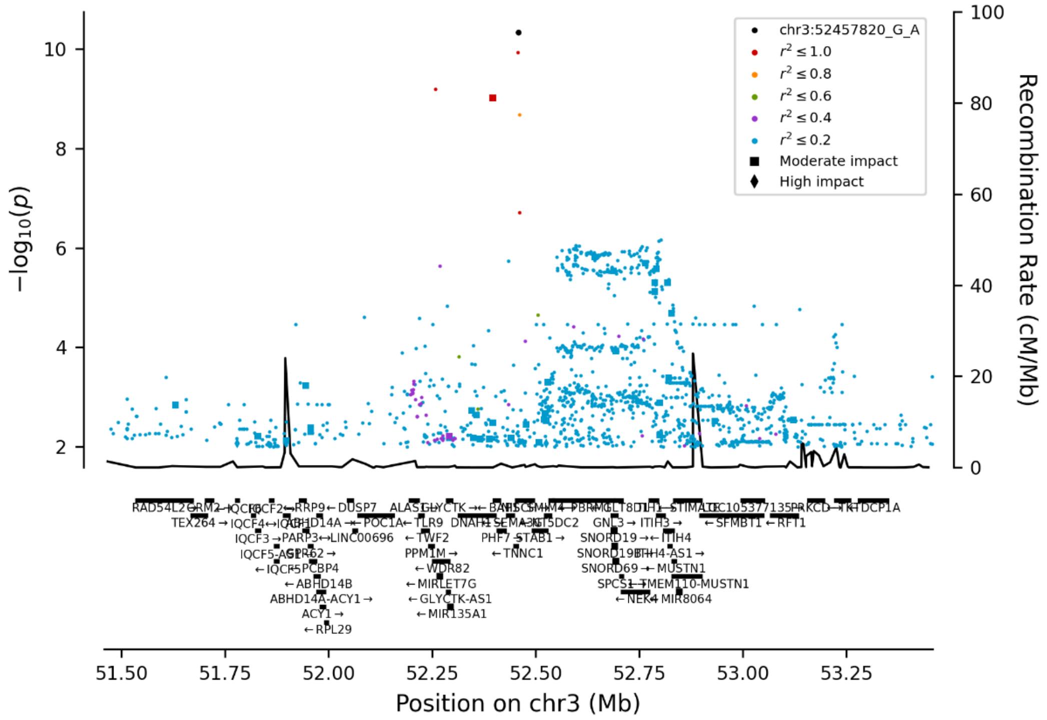

Supplementary locus plot 7: Locus plot of novel variant, rs12629472, with ± 500Kb flanking region, the color code represnt r^2 which is a measure (LD) of the lead variant with surrounding variants.

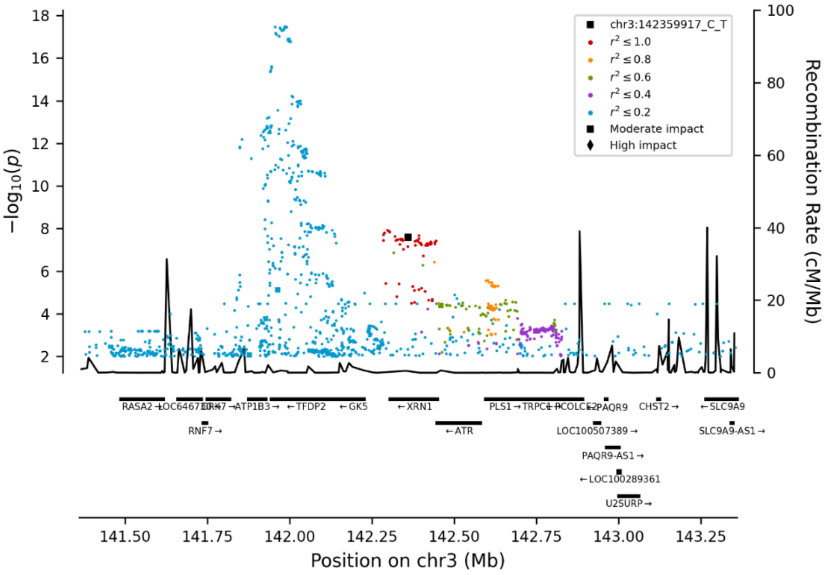

Supplementary locus plot 8: Locus plot of novel variant, rs73238159, with ± 500Kb flanking region, the color code represnt r^2 which is a measure (LD) of the lead variant with surrounding variants.

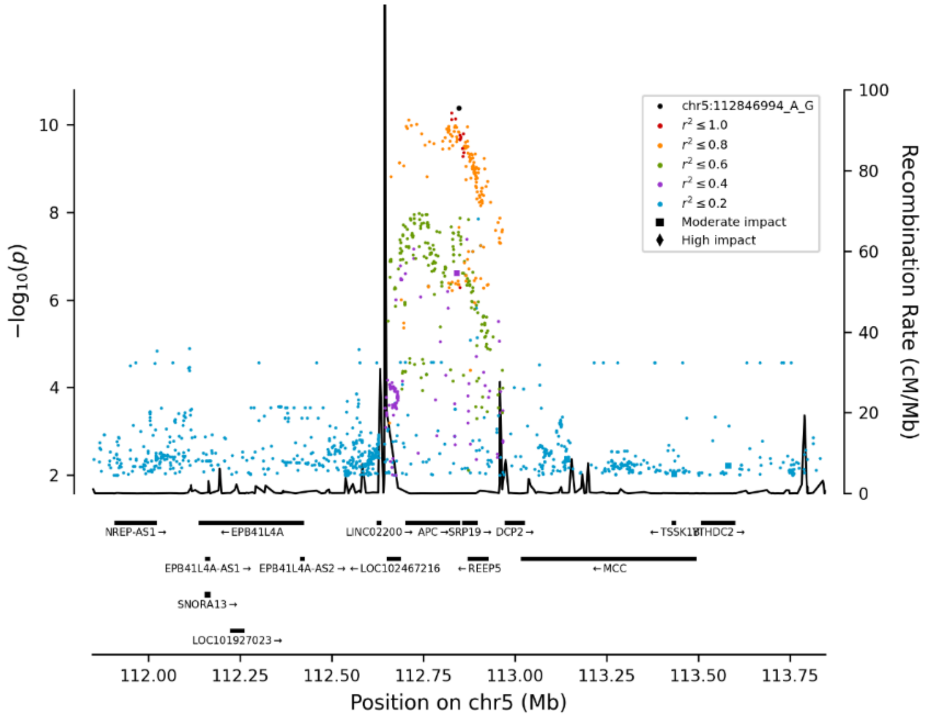

Supplementary locus plot 9: Locus plot of novel variant, rs448162, with ± 500Kb flanking region, the color code represnt r^2 which is a measure (LD) of the lead variant with surrounding variants.

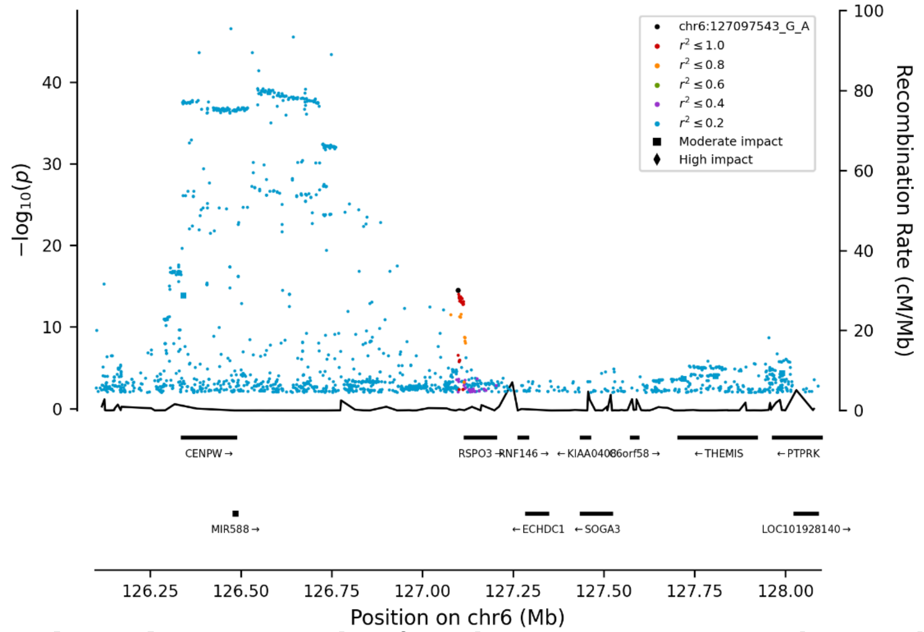

Supplementary locus plot 10: Locus plot of novel variant, rs12524625, with ± 500Kb flanking region, the color code represnt r^2 which is a measure (LD) of the lead variant with surrounding variants.

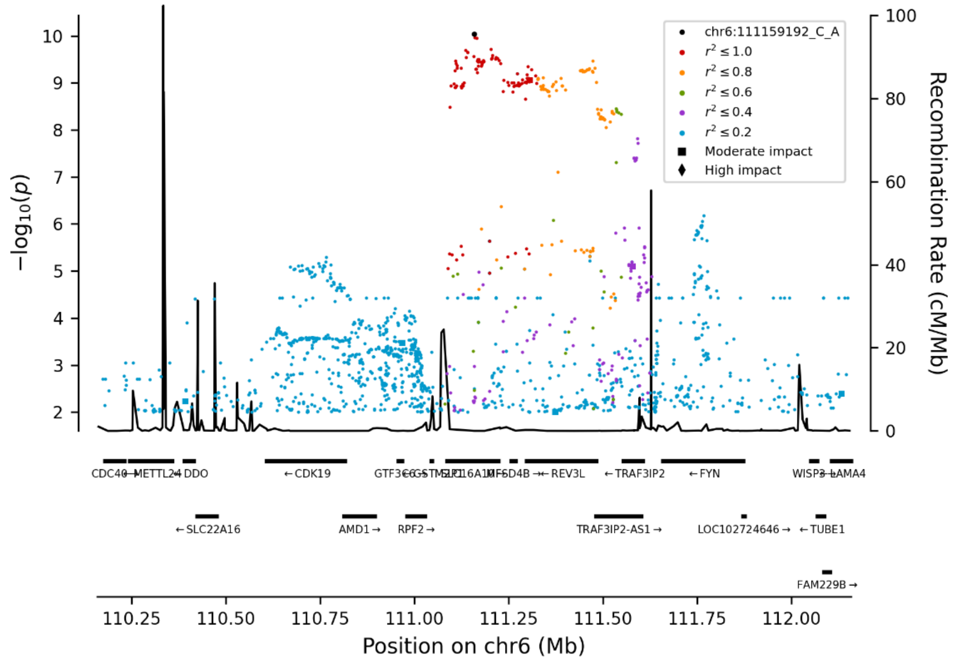

Supplementary locus plot 11: Locus plot of novel variant, rs449308, with ± 500Kb flanking region, the color code represnt r^2 which is a measure (LD) of the lead variant with surrounding variants.

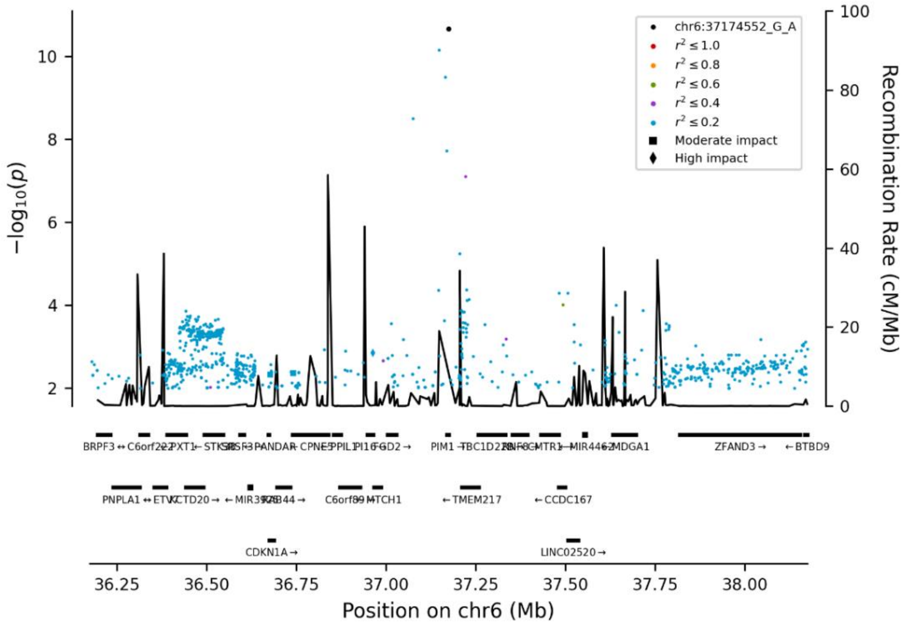

Supplementary locus plot 12: Locus plot of novel variant, rs180819997, with ± 500Kb flanking region, the color code represnt r^2 which is a measure (LD) of the lead variant with surrounding variants.

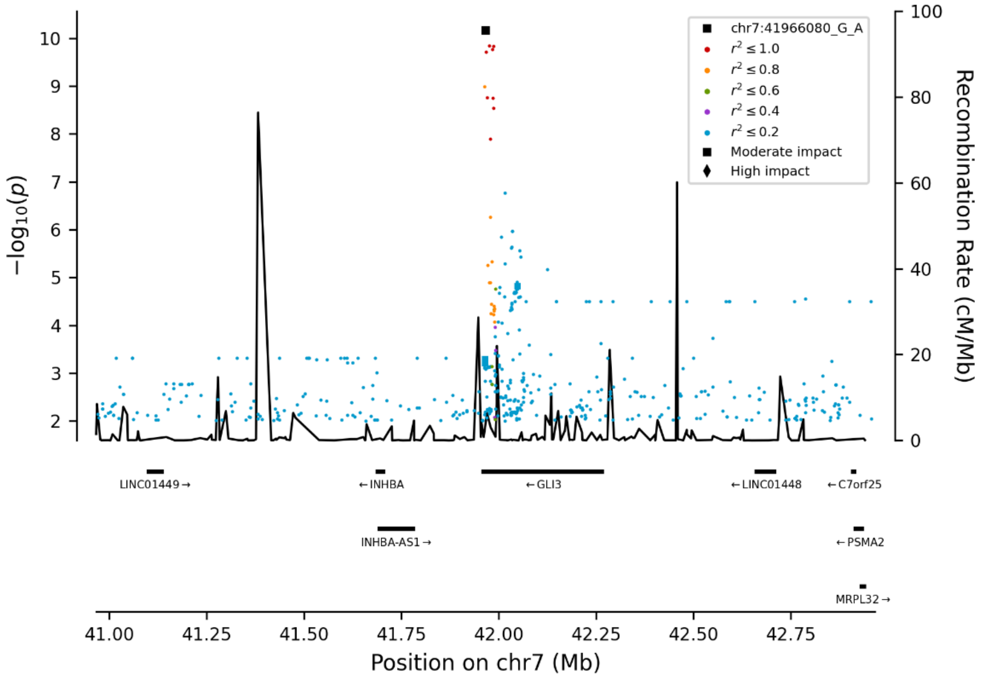

Supplementary locus plot 13: Locus plot of novel variant, rs929387, with ± 500Kb flanking region, the color code represnt r^2 which is a measure (LD) of the lead variant with surrounding variants.

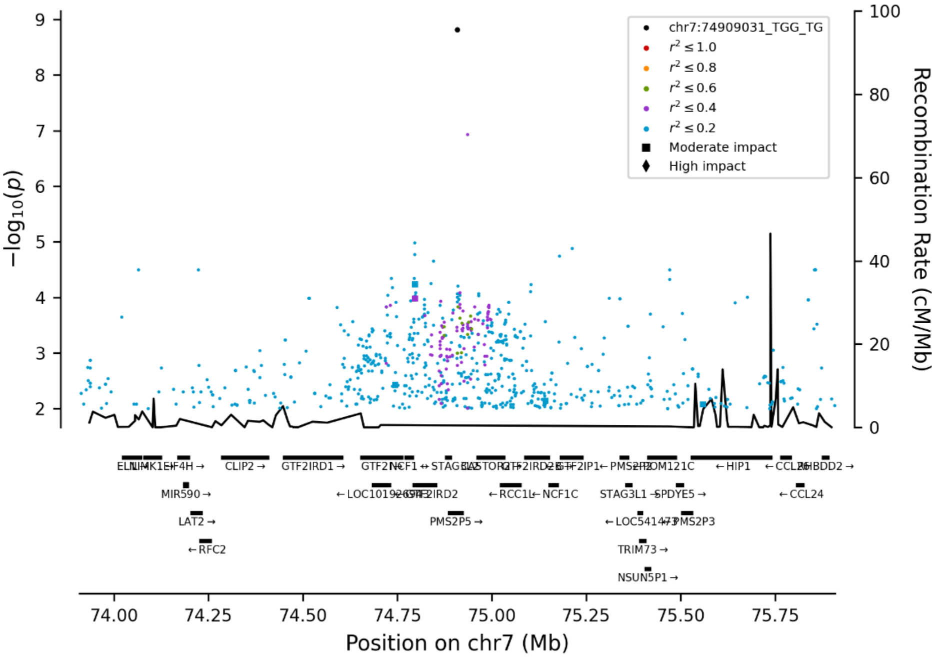

Supplementary locus plot 14: Locus plot of novel variant, rs1372063030, with ± 500Kb flanking region, the color code represnt r^2 which is a measure (LD) of the lead variant with surrounding variants.

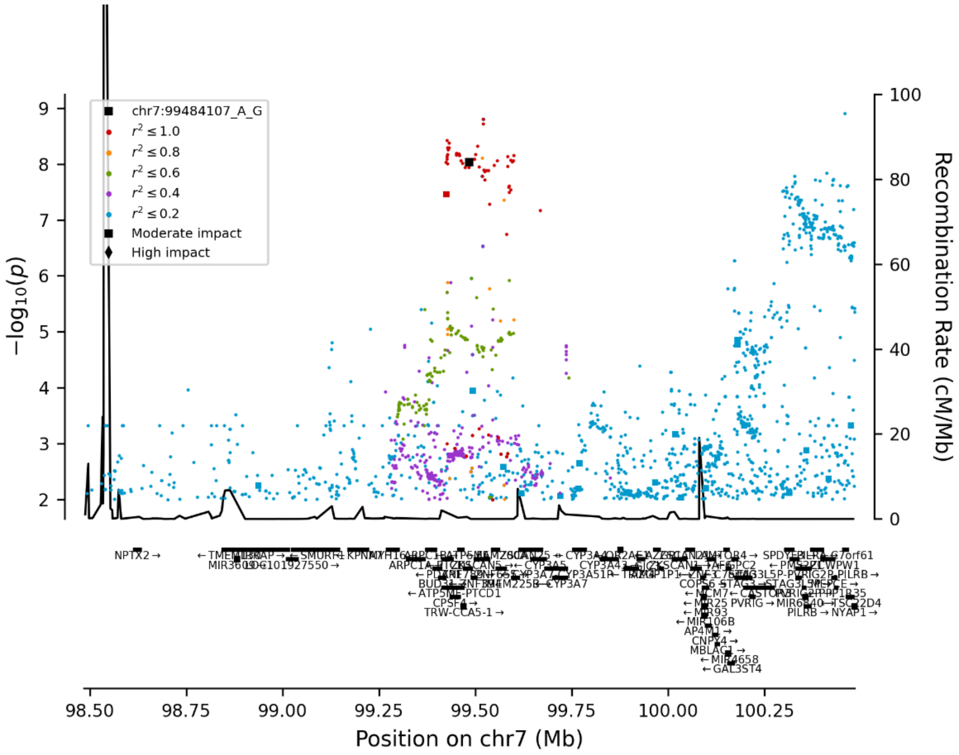

Supplementary locus plot 15: Locus plot of novel variant, rs6962772, with ± 500Kb flanking region, the color code represnt r^2 which is a measure (LD) of the lead variant with surrounding variants.

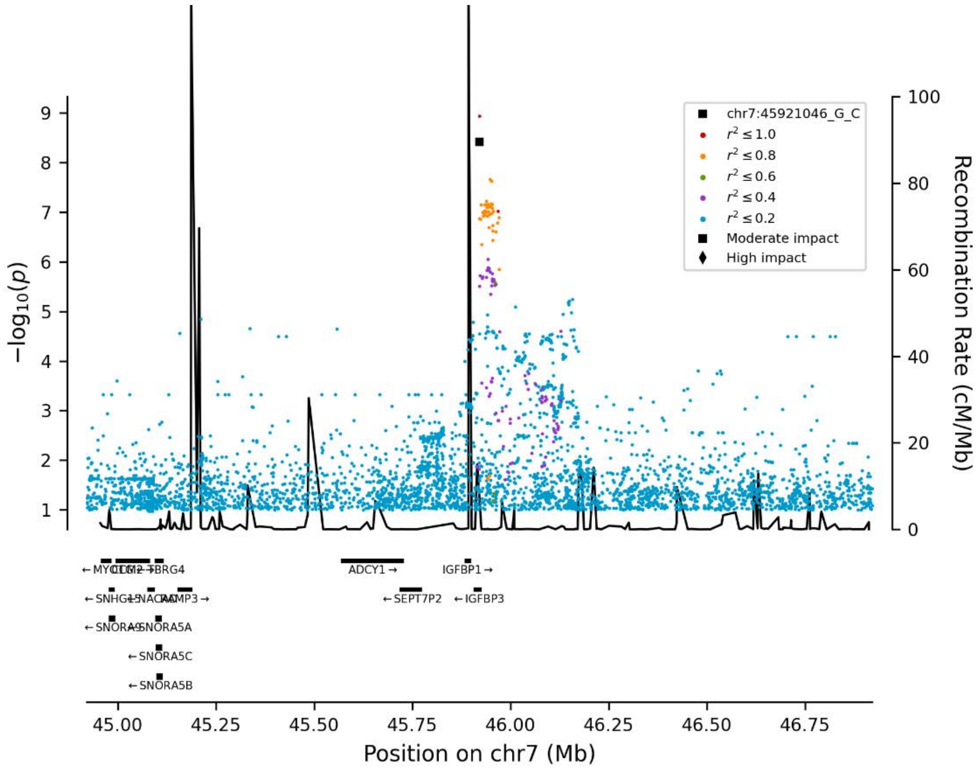

Supplementary locus plot 16: Locus plot of novel variant, chr7_45921046_G_C, with ± 500Kb flanking region, the color code represnt r^2 which is a measure (LD) of the lead variant with surrounding variants.

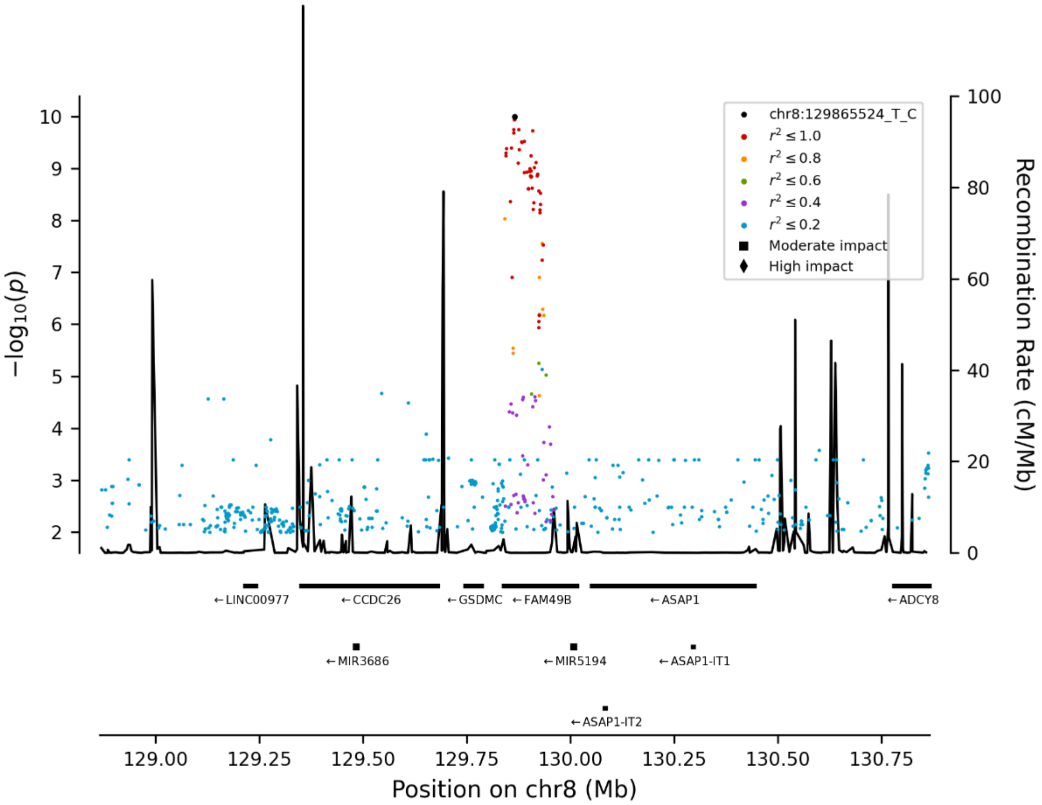

Supplementary locus plot 17: Locus plot of novel variant, rs384752, with ± 500Kb flanking region, the color code represnt r^2 which is a measure (LD) of the lead variant with surrounding variants.

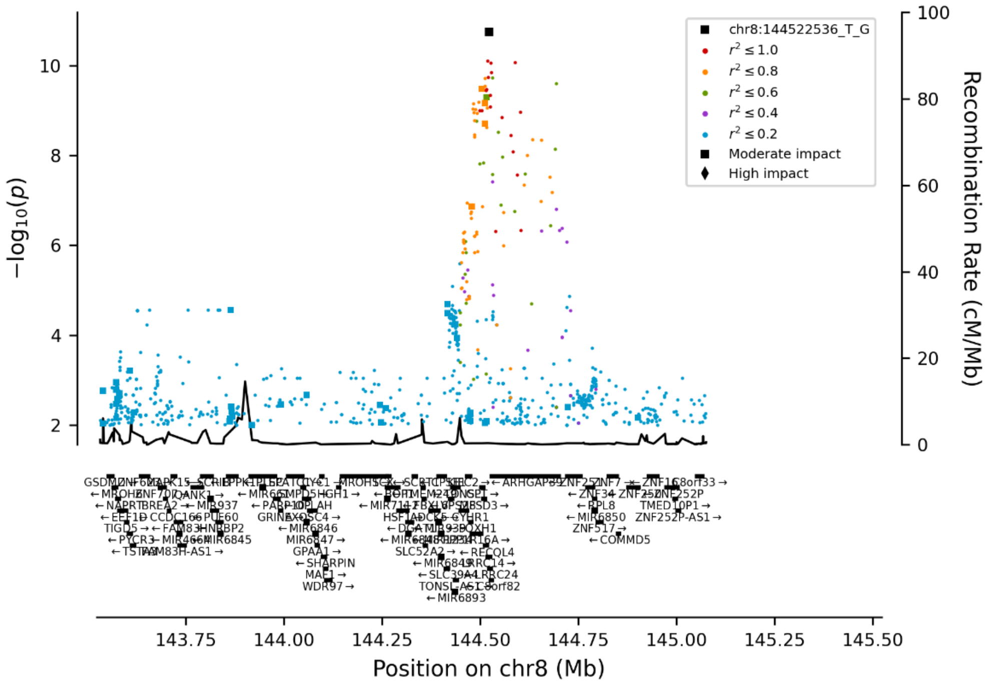

Supplementary locus plot 18: Locus plot of novel variant, rs13277542, with ± 500Kb flanking region, the color code represnt r^2 which is a measure (LD) of the lead variant with surrounding variants.

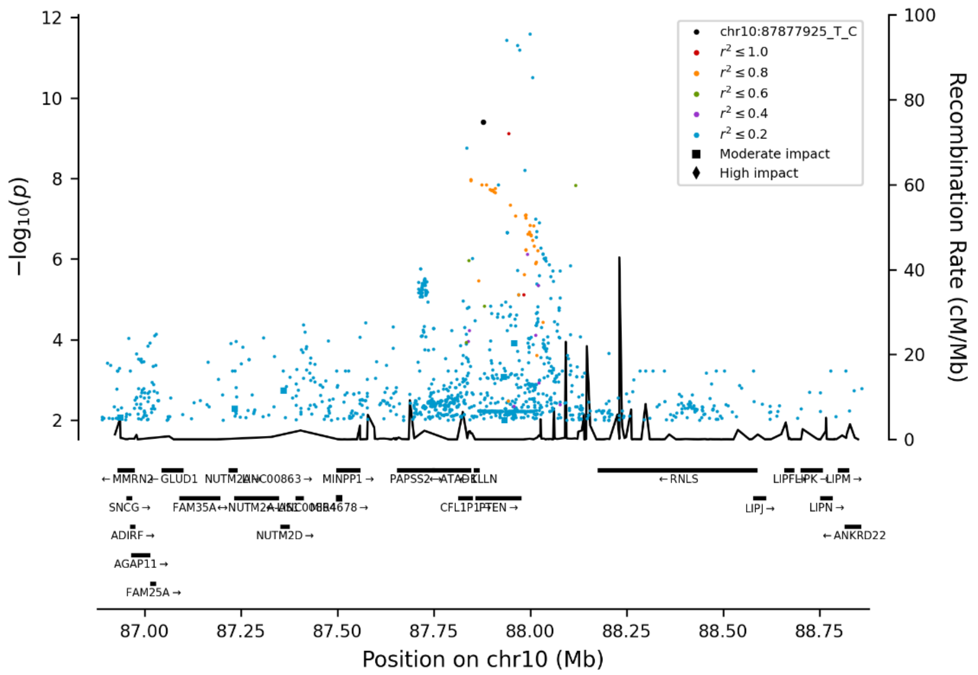

Supplementary locus plot 19: Locus plot of novel variant, rs72814160, with ± 500Kb flanking region, the color code represnt r^2 which is a measure (LD) of the lead variant with surrounding variants.

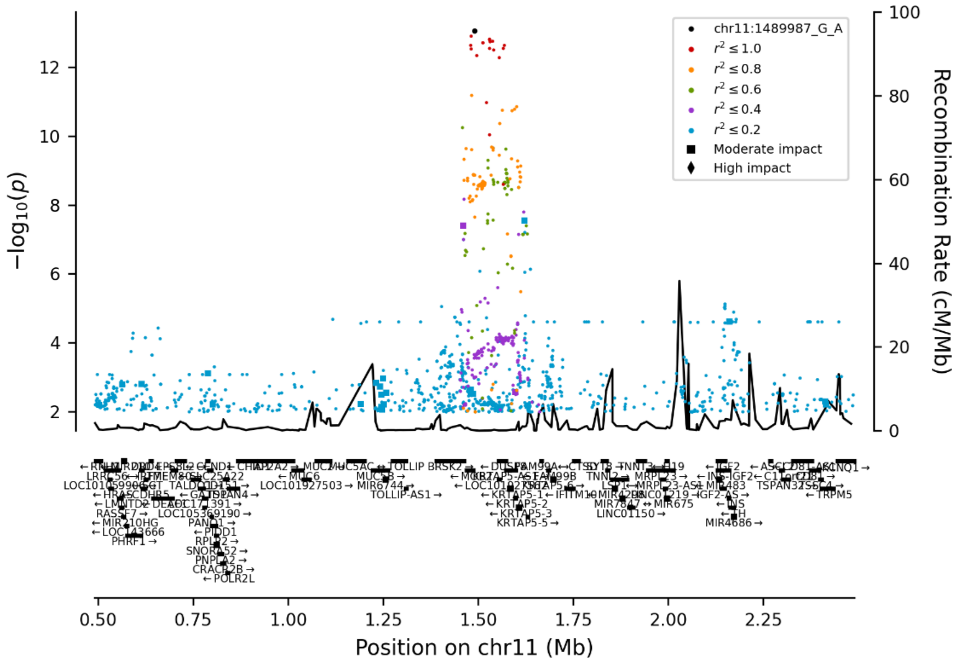

Supplementary locus plot 20: Locus plot of novel variant, rs2334552, with ± 500Kb flanking region, the color code represnt r^2 which is a measure (LD) of the lead variant with surrounding variants.

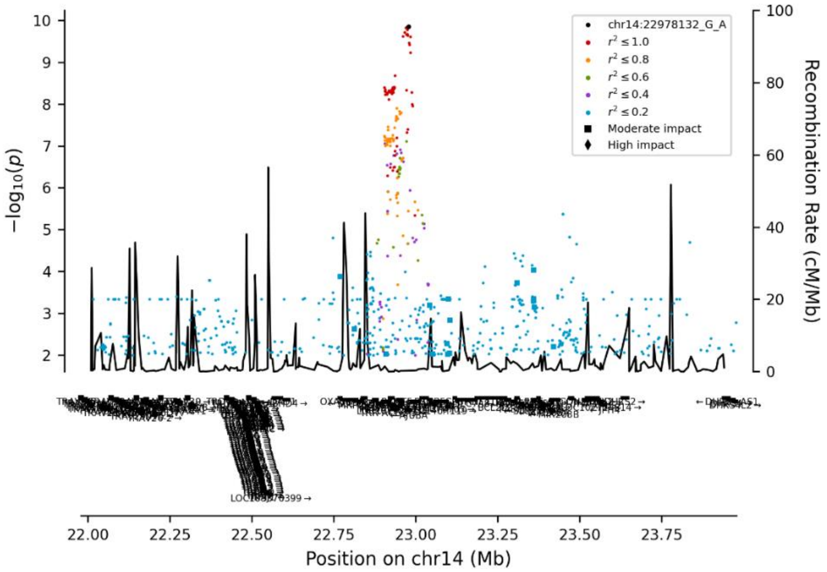

Supplementary locus plot 21: Locus plot of novel variant, rs6572891, with ± 500Kb flanking region, the color code represnt r^2 which is a measure (LD) of the lead variant with surrounding variants.

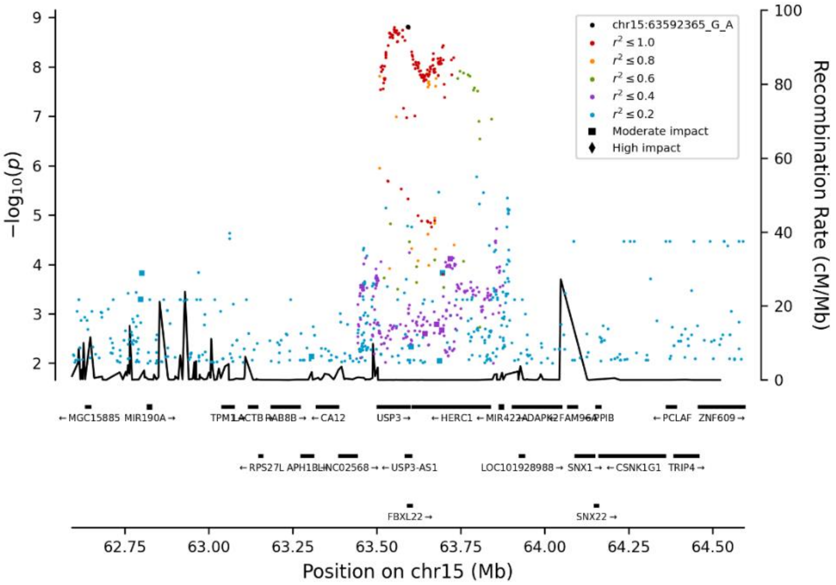

Supplementary locus plot 22: Locus plot of novel variant, rs76115381, with ± 500Kb flanking region, the color code represnt r^2 which is a measure (LD) of the lead variant with surrounding variants.

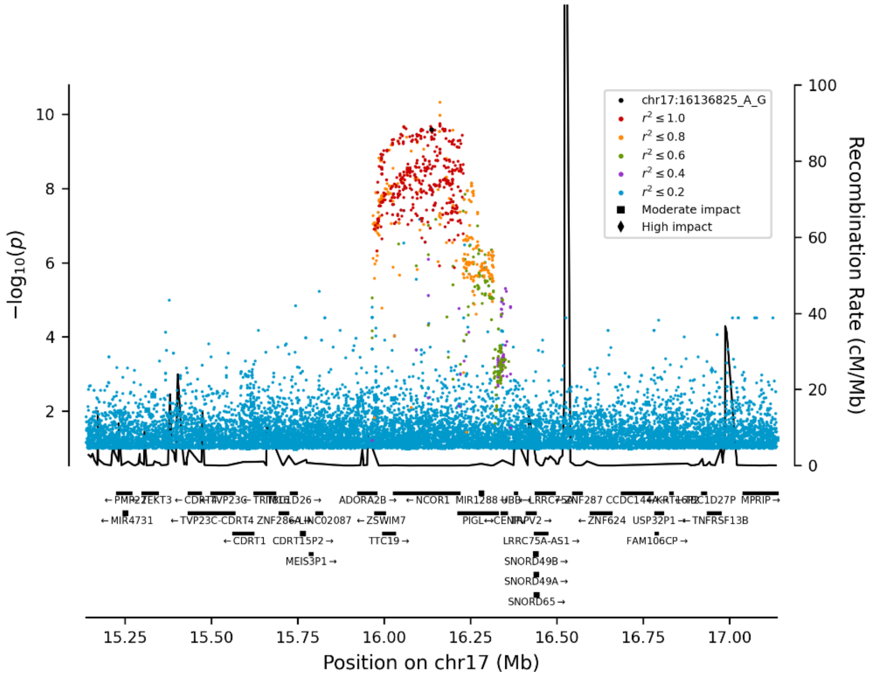

Supplementary locus plot 23: Locus plot of novel variant, rs1986040, with ± 500Kb flanking region, the color code represnt r[2] which is a measure (LD) of the lead variant with surrounding variants. This association is mainly driven by UKB sample where a few stronger than ‚rs1986040‘ are singelton variant from UKB, therefore we report association of rs1986040 as this variant is also found in other populations.

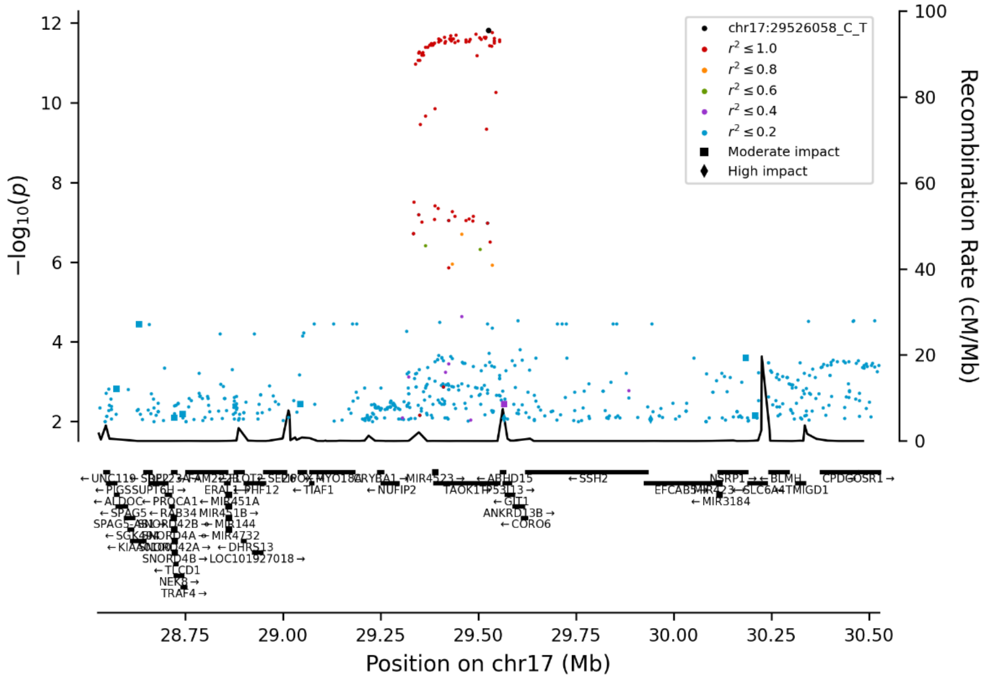

Supplementary locus plot 24: Locus plot of novel variant, rs35260328, with ± 500Kb flanking region, the color code represnt r[2] which is a measure (LD) of the lead variant with surrounding variants.

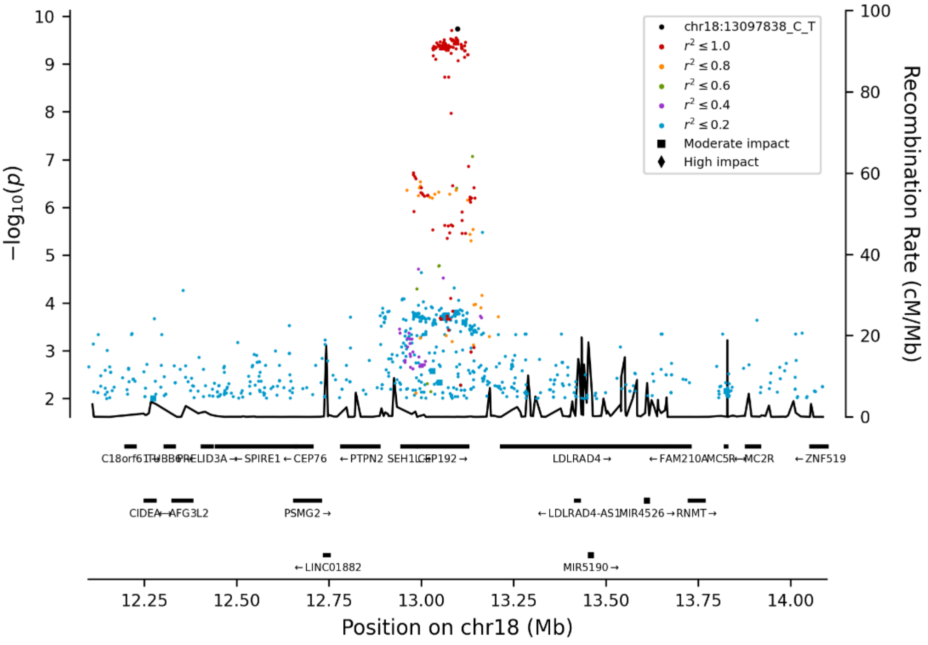

Supplementary locus plot 25: Locus plot of novel variant, rs150893238, with ± 500Kb flanking region, the color code represnt r^2 which is a measure (LD) of the lead variant with surrounding variants.

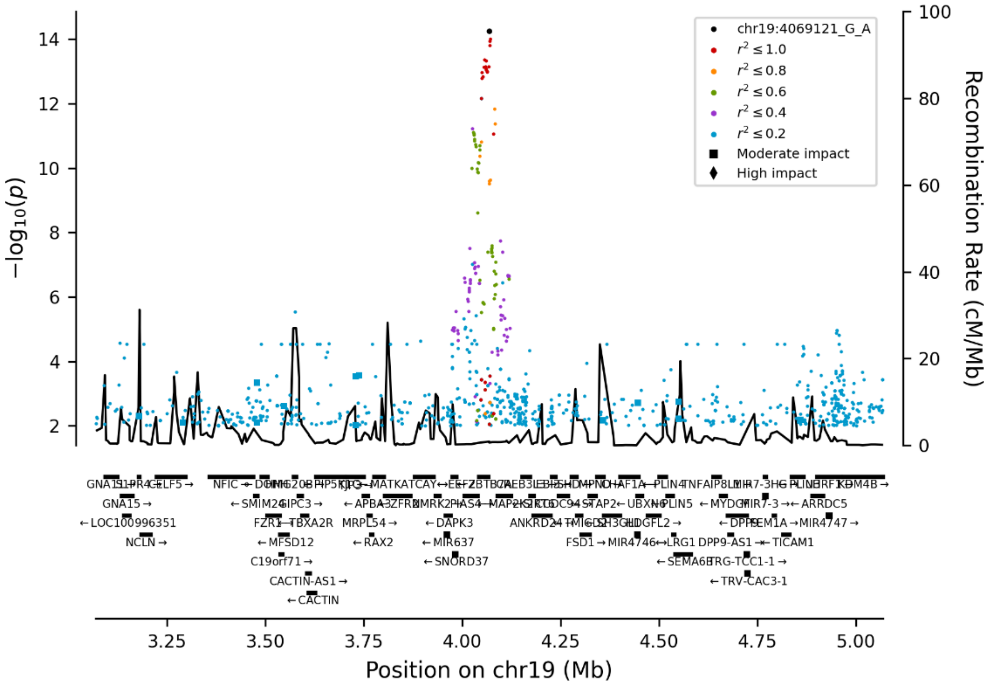

Supplementary locus plot 26: Locus plot of novel variant, rs7254272, with ± 500Kb flanking region, the color code represnt r^2 which is a measure (LD) of the lead variant with surrounding variants.

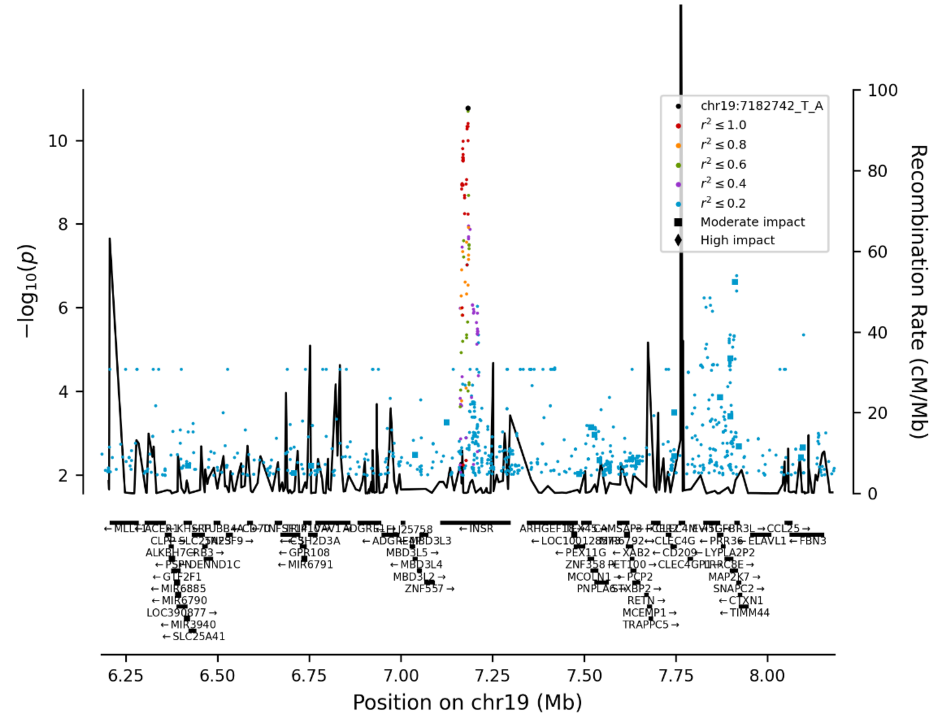

Supplementary locus plot 27: Locus plot of novel variant, rs8108622, with ± 500Kb flanking region, the color code represnt r^2 which is a measure (LD) of the lead variant with surrounding variants.

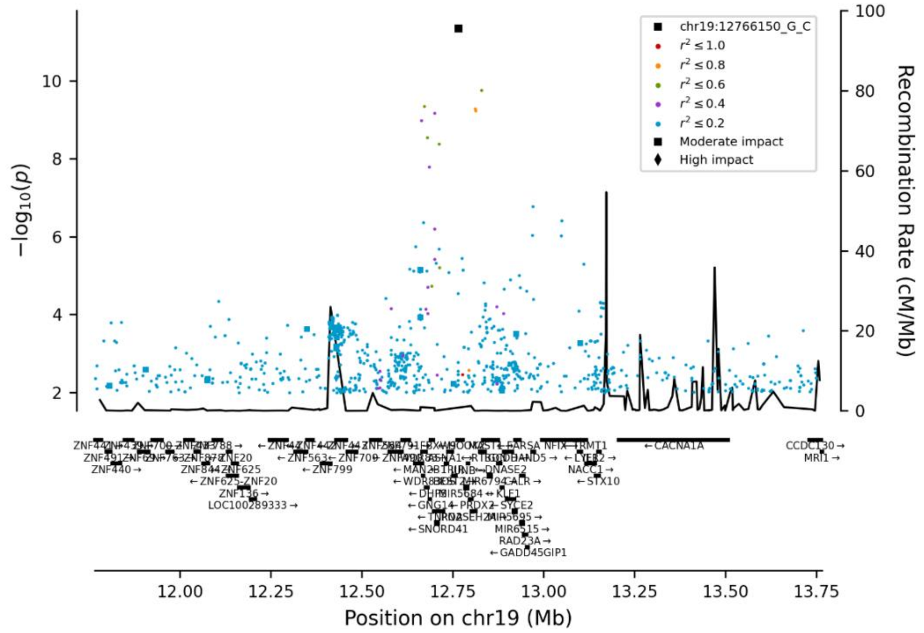

Supplementary locus plot 28: Locus plot of novel variant, rs897804, with ± 500Kb flanking region, the color code represnt r^2 which is a measure (LD) of the lead variant with surrounding variants.

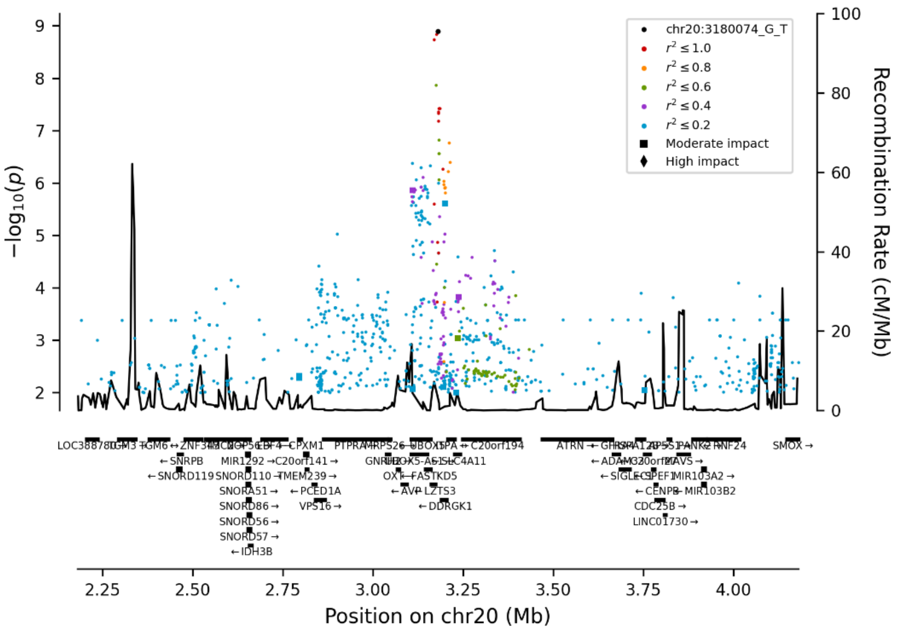

Supplementary locus plot 29: Locus plot of novel variant, rs6051621, with ± 500Kb flanking region, the color code represnt r^2 which is a measure (LD) of the lead variant with surrounding variants.

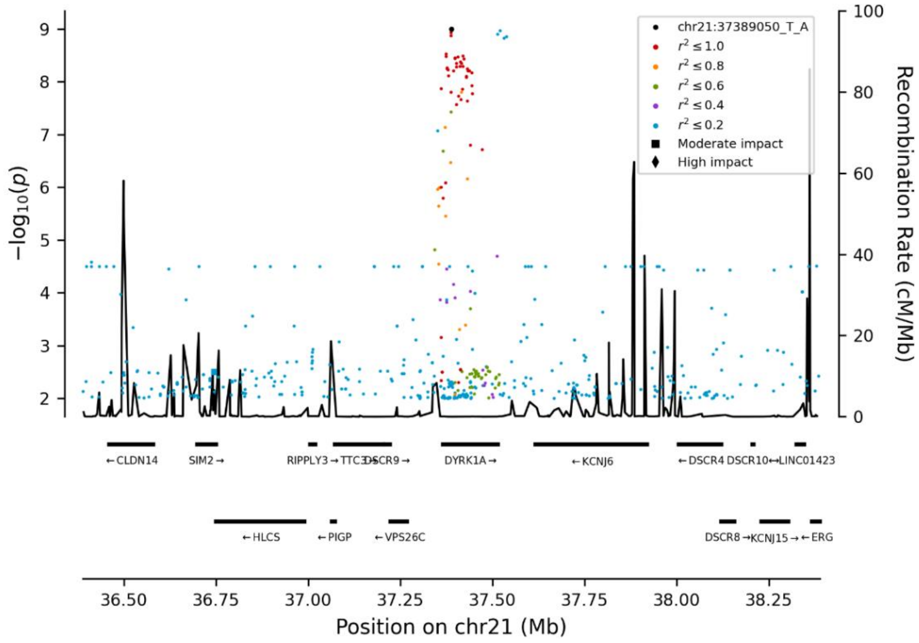

Supplementary locus plot 30: Locus plot of novel variant, rs9979506, with ± 500Kb flanking region, the color code represnt r^2 which is a measure (LD) of the lead variant with surrounding variants.

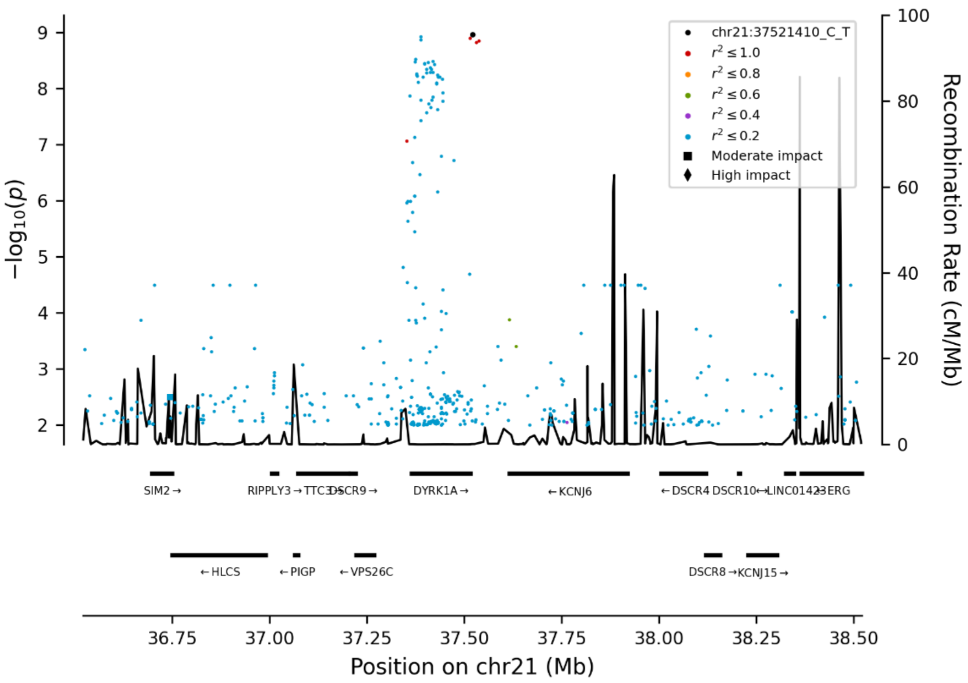

Supplementary locus plot 31: Locus plot of novel variant, rs17814794, with ± 500Kb flanking region, the color code represnt r^2 which is a measure (LD) of the lead variant with surrounding variants.

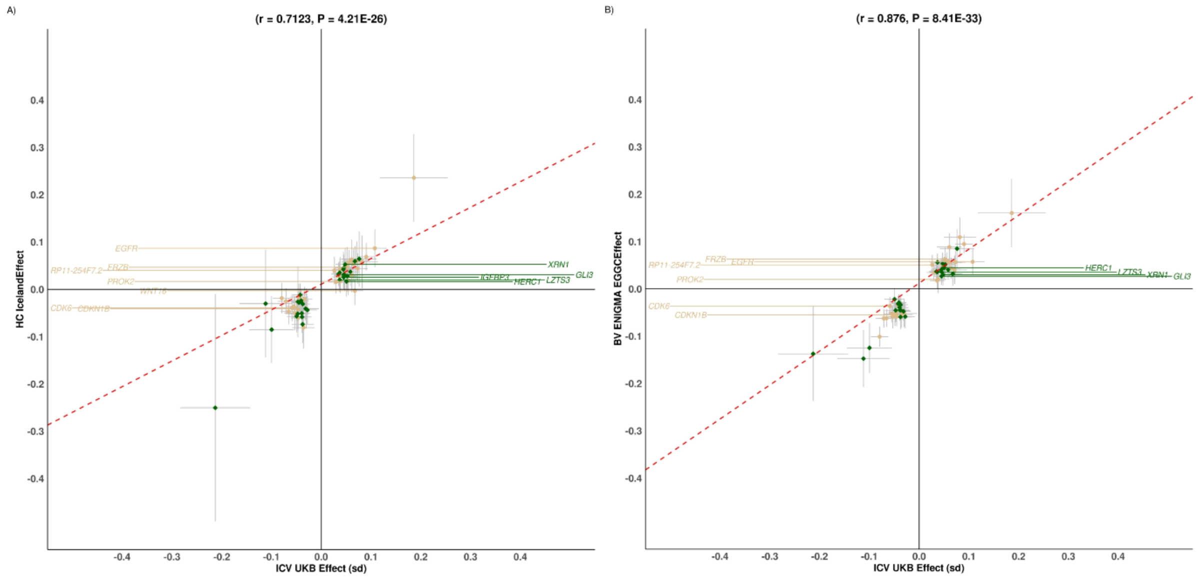

Supplementary Figure 32: Comparison of effect sizes of 64 ICV variants on ICV vs HC or ICV in different samples from UKB, Iceland, and ENIGMA+EGGC. The green highlighted are novel ICV variants while light brown are known ICV variants, implicated genes are highlighted, and correlation estimate of effect size on ICV vs HC or ICV is given on the top.

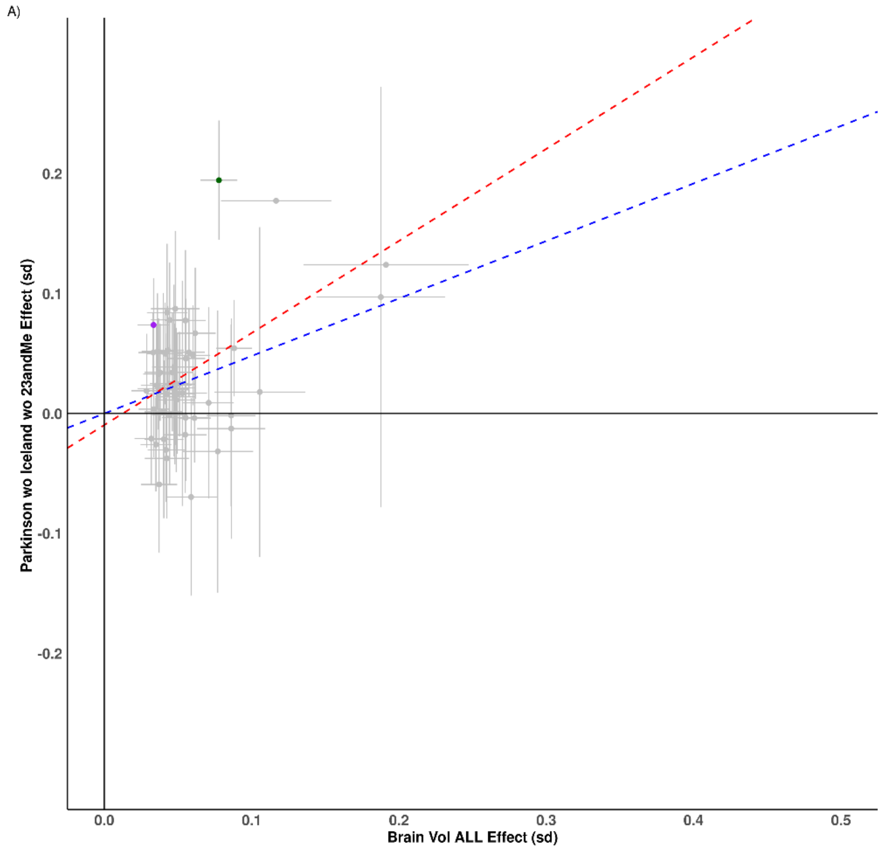

Supplementary figure 33: Effect vs effect plots of variants associated with intracranial volume (*N* = 64) vs Parkinson disease. On *X-axis* are effect size for intracranial volume meta-analysis (Iceland + UKB + ENIGMA + EGGC) and on *y-axis* for Parkinson disease. All effects are plotted for alleles with increasing intracranial volume. Blue line represents slope estimated through inverse variance weighted method (IVW), and red line MR Egger method including intercept. Green dots represent conventional GWAS associations ($P < 5.0\times10^{-8}$), purple are Bonferroni significant associations ($P < 0.05/64 = 0.00078$).

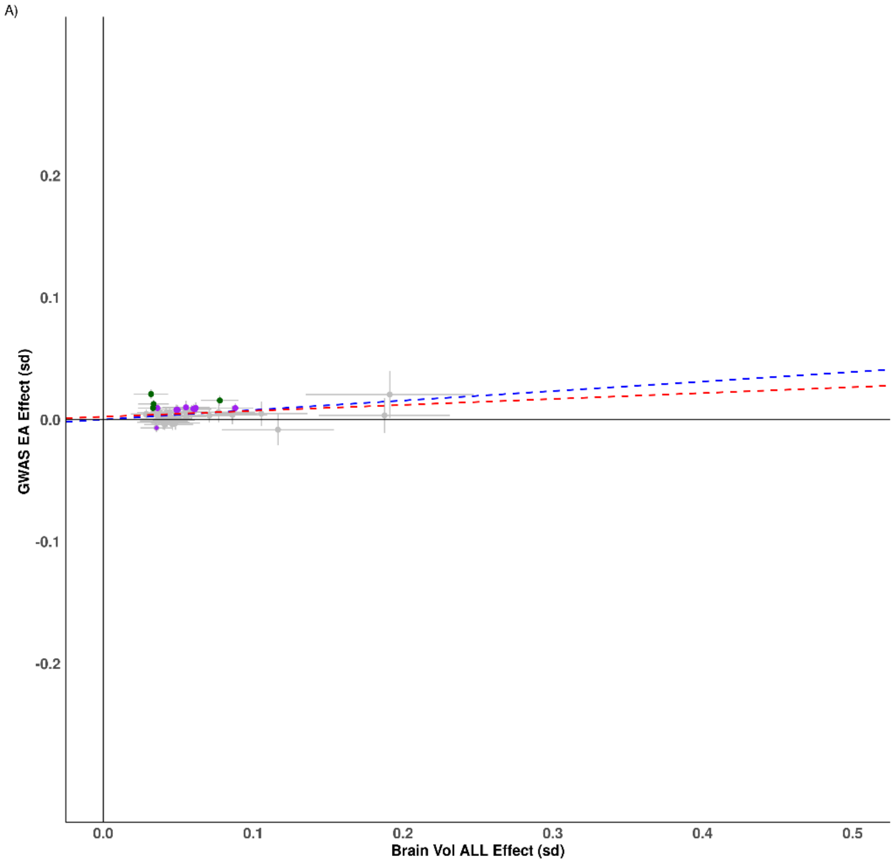

Supplementary figure 34: Effect vs effect plots of variants associated with intracranial volume (*N* = 64) vs educational attainment. On *X-axis* are effect size for intracranial volume meta-analysis (Iceland + UKB + ENIGMA + EGGC) and on *y-axis* for educational attainment. All effects are plotted for alleles with increasing intracranial volume. Blue line represents slope estimated through inverse variance weighted method (IVW), and red line MR Egger method including intercept. Green dots represent conventional GWAS associations ($P < 5.0\times10^{-8}$), purple are Bonferroni significant associations ($P < 0.05/64 = 0.00078$).

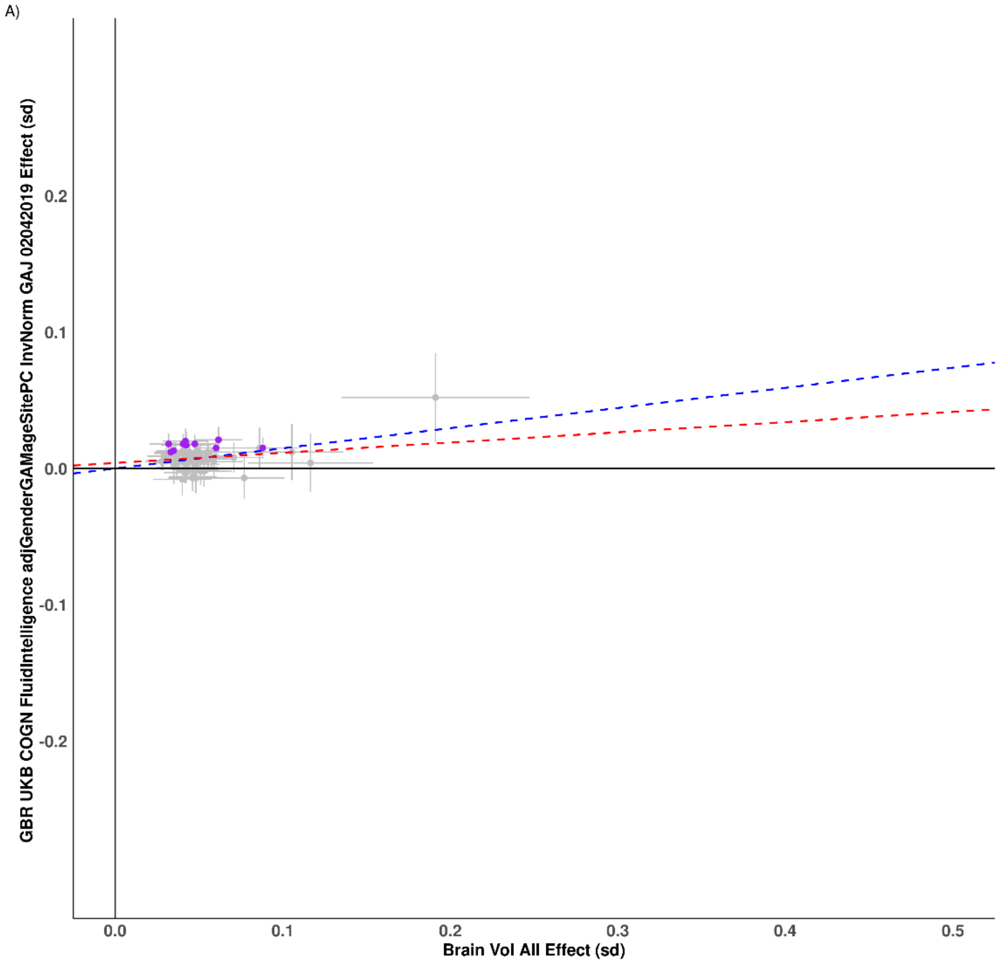

Supplementary figure 35: Effect vs effect plots of variants associated with intracranial volume (*N* = 64) vs fluid intelligence. On *X-axis* are effect size for intracranial volume meta-analysis (Iceland + UKB + ENIGMA + EGGC) and on *y-axis* for fluid intelligence. All effects are plotted for alleles with increasing intracranial volume. Blue line represents slope estimated through inverse variance weighted method (IVW), and red line MR Egger method including intercept. Green dots represent conventional GWAS associations ($P < 5.0\times10^{-8}$), purple are Bonferroni significant associations ($P < 0.05/64 = 0.00078$).

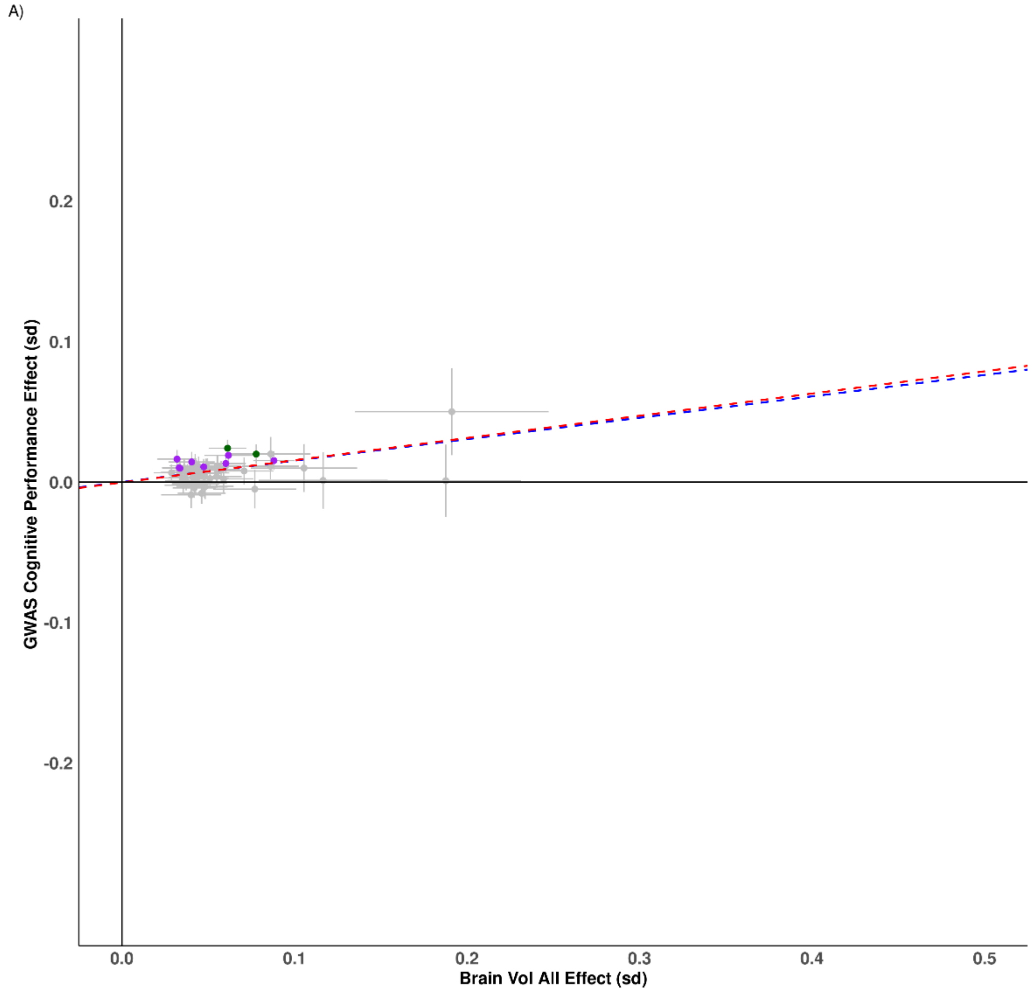

Supplementary figure 36: Effect vs effect plots of variants associated with intracranial volume (*N* = 64) vs cognitive performance. On *X-axis* are effect size for intracranial volume meta-analysis (Iceland + UKB + ENIGMA + EGGC) and on *y-axis* for cognitive performance. All effects are plotted for alleles with increasing intracranial volume. Blue line represents slope estimated through inverse variance weighted method (IVW), and red line MR Egger method including intercept. Green dots represent conventional GWAS associations ($P < 5.0\times10^{-8}$), purple are Bonferroni significant associations ($P < 0.05/64 = 0.00078$).

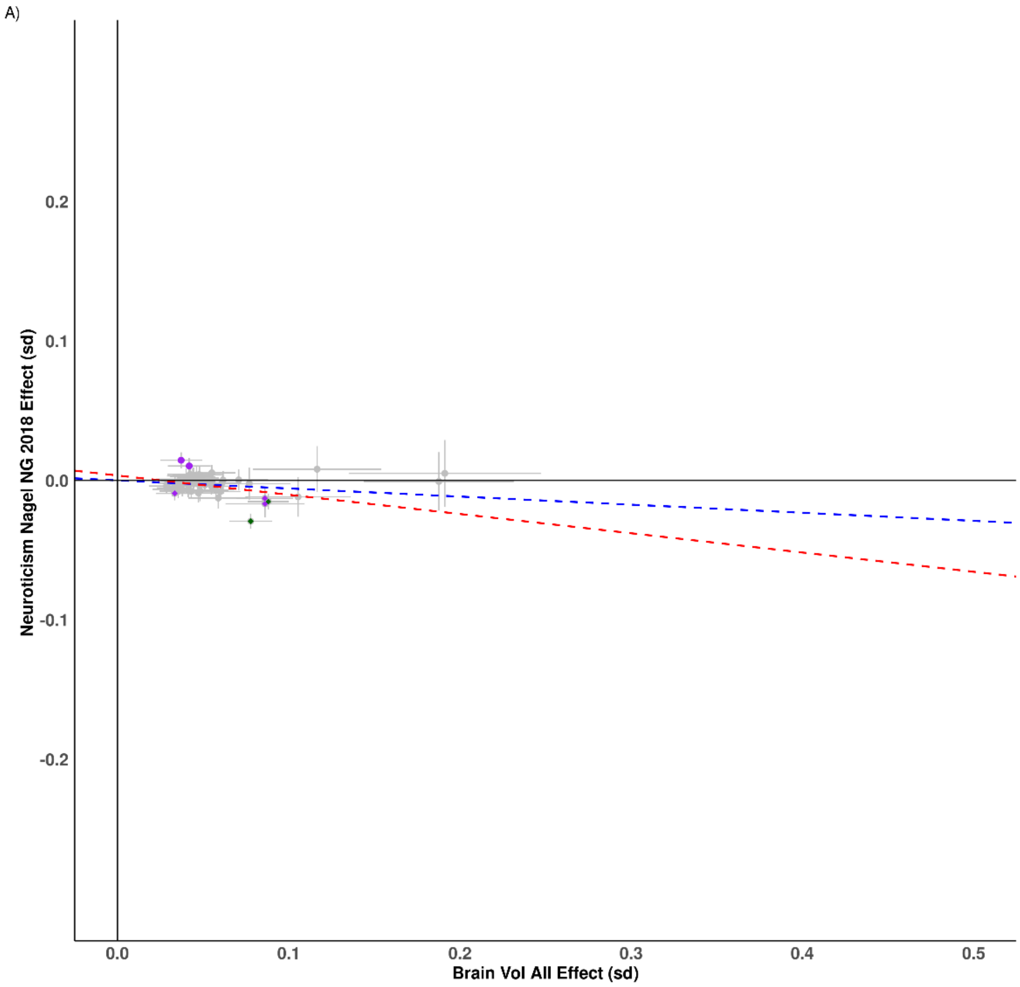

Supplementary figure 37: Effect vs effect plots of variants associated with intracranial volume (*N* = 64) vs neuroticism. On *X-axis* are effect size for intracranial volume meta-analysis (Iceland + UKB + ENIGMA + EGGC) and on *y-axis* for neuroticism. All effects are plotted for alleles with increasing intracranial volume. Blue line represents slope estimated through inverse variance weighted method (IVW), and red line MR Egger method including intercept. Green dots represent conventional GWAS associations ($P < 5.0\times10^{-8}$), purple are Bonferroni significant associations ($P < 0.05/64 = 0.00078$).

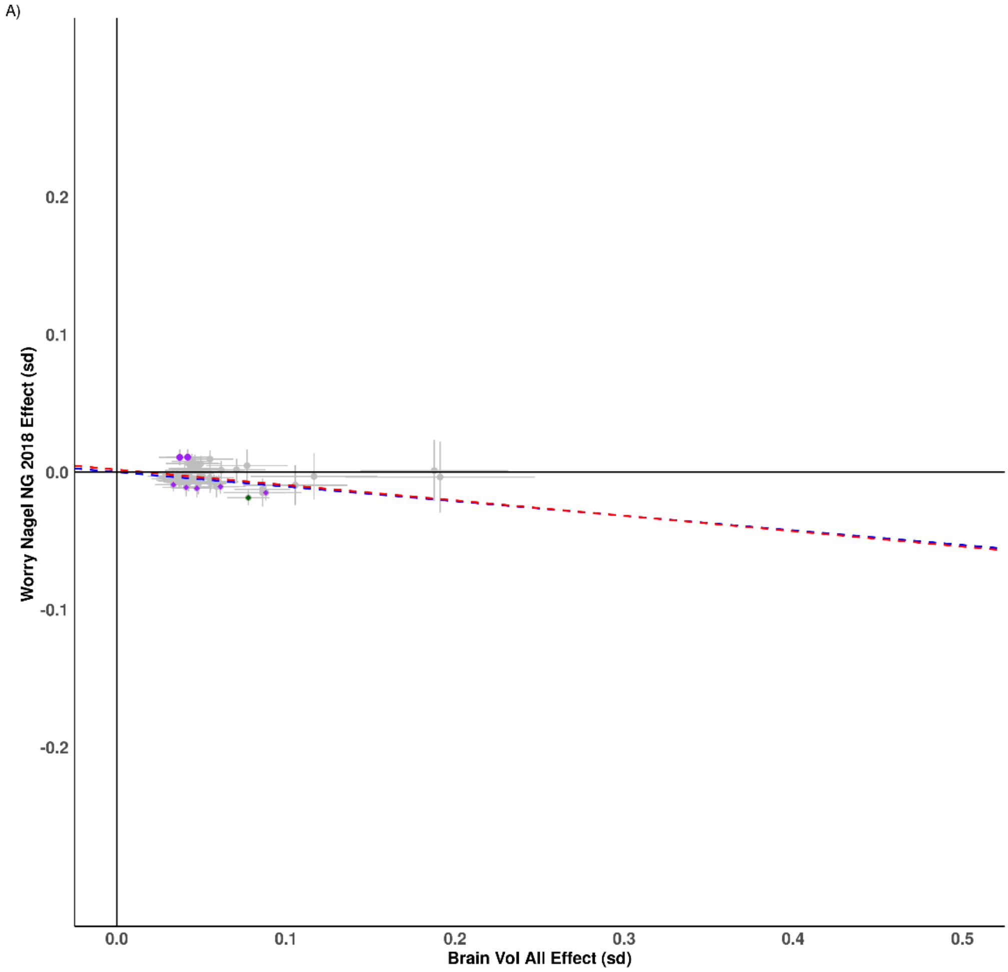

Supplementary figure 38: Effect vs effect plots of variants associated with intracranial volume (*N* = 64) vs worry personality. On *X-axis* are effect size for intracranial volume meta-analysis (Iceland + UKB + ENIGMA + EGGC) and on *y-axis* for worry personality. All effects are plotted for alleles with increasing intracranial volume. Blue line represents slope estimated through inverse variance weighted method (IVW), and red line MR Egger method including intercept. Green dots represent conventional GWAS associations ($P < 5.0\times10^{-8}$), purple are Bonferroni significant associations ($P < 0.05/64 = 0.00078$).

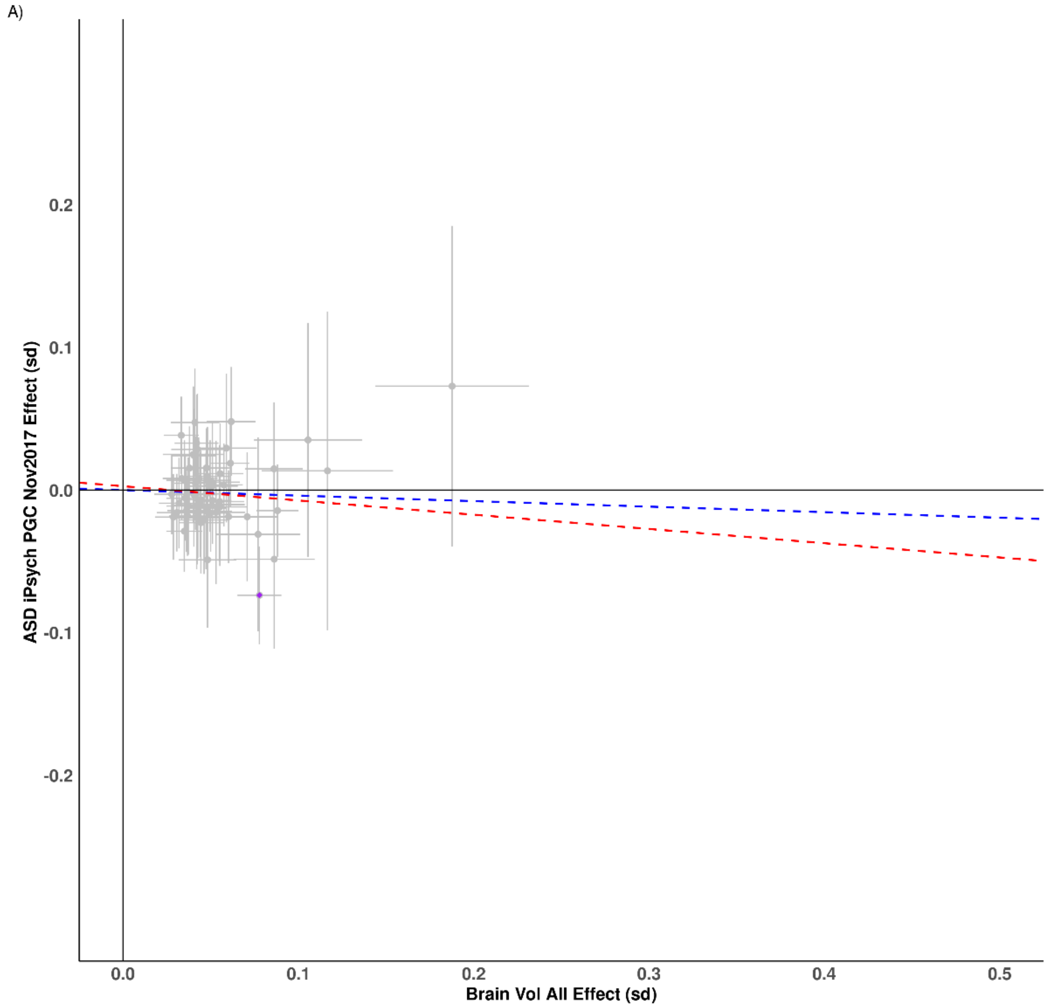

Supplementary figure 39: Effect vs effect plots of variants associated with intracranial volume (*N* = 64) vs Autism. On *X-axis* are effect size for intracranial volume meta-analysis (Iceland + UKB + ENIGMA + EGGC) and on *y-axis* for Autism. All effects are plotted for alleles with increasing intracranial volume. Blue line represents slope estimated through inverse variance weighted method (IVW), and red line MR Egger method including intercept. Green dots represent conventional GWAS associations ($P < 5.0{\times}10^{-8}$), purple are Bonferroni significant associations ($P < 0.05/64 = 0.00078$).

A)

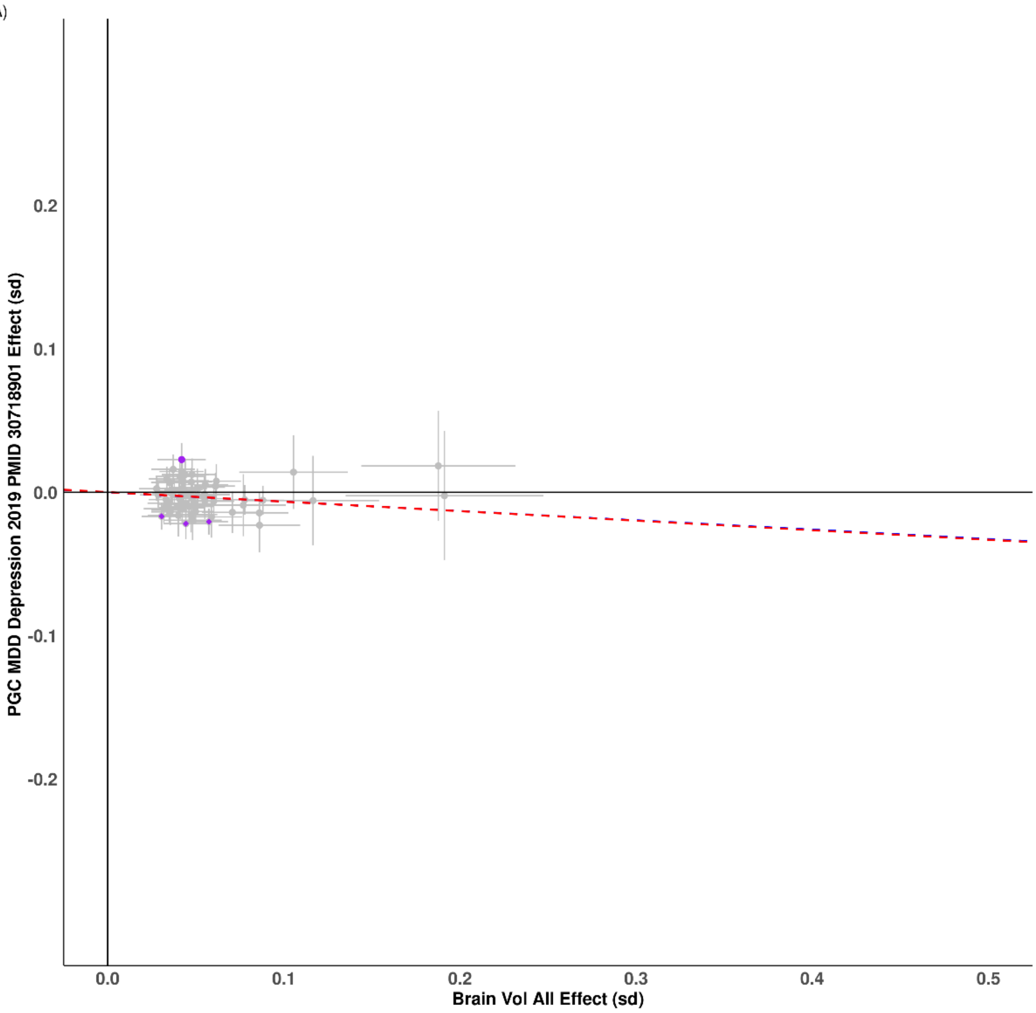

Supplementary figure 40: Effect vs effect plots of variants associated with intracranial volume (*N* = 64) vs depression. On *X-axis* are effect size for intracranial volume meta-analysis (Iceland + UKB + ENIGMA + EGGC) and on *y-axis* for depression. All effects are plotted for alleles with increasing intracranial volume. Blue line represents slope estimated through inverse variance weighted method (IVW), and red line MR Egger method including intercept. Green dots represent conventional GWAS associations ($P < 5.0{\times}10^{-8}$), purple are Bonferroni significant associations ($P < 0.05/64 = 0.00078$).

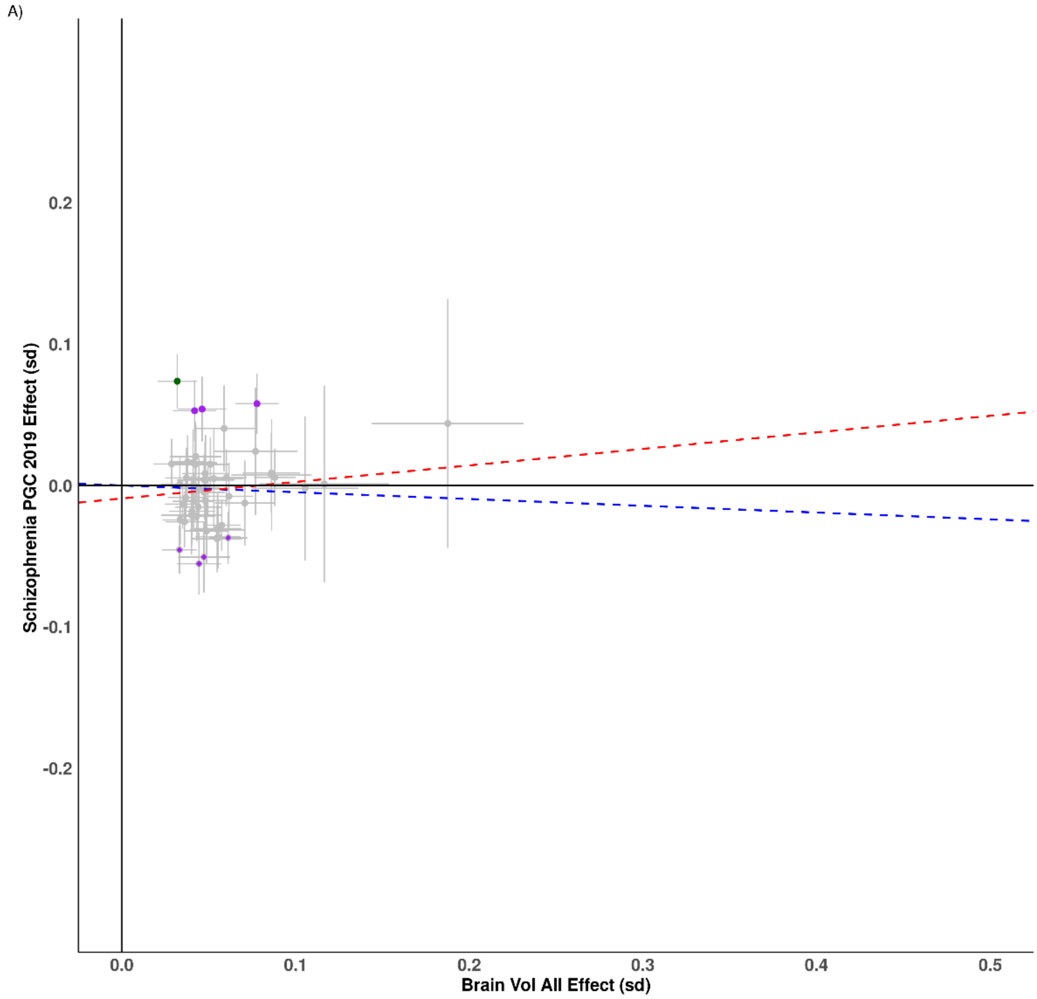

Supplementary figure 41: Effect vs effect plots of variants associated with intracranial volume (*N* = 64) vs Schizophrenia. On *X-axis* are effect size for intracranial volume meta-analysis (Iceland + UKB + ENIGMA + EGGC) and on *y-axis* for Schizophrenia. All effects are plotted for alleles with increasing intracranial volume. Blue line represents slope estimated through inverse variance weighted method (IVW), and red line MR Egger method including intercept. Green dots represent conventional GWAS associations ($P < 5.0\times10^{-8}$), purple are Bonferroni significant associations ($P < 0.05/64 = 0.00078$).

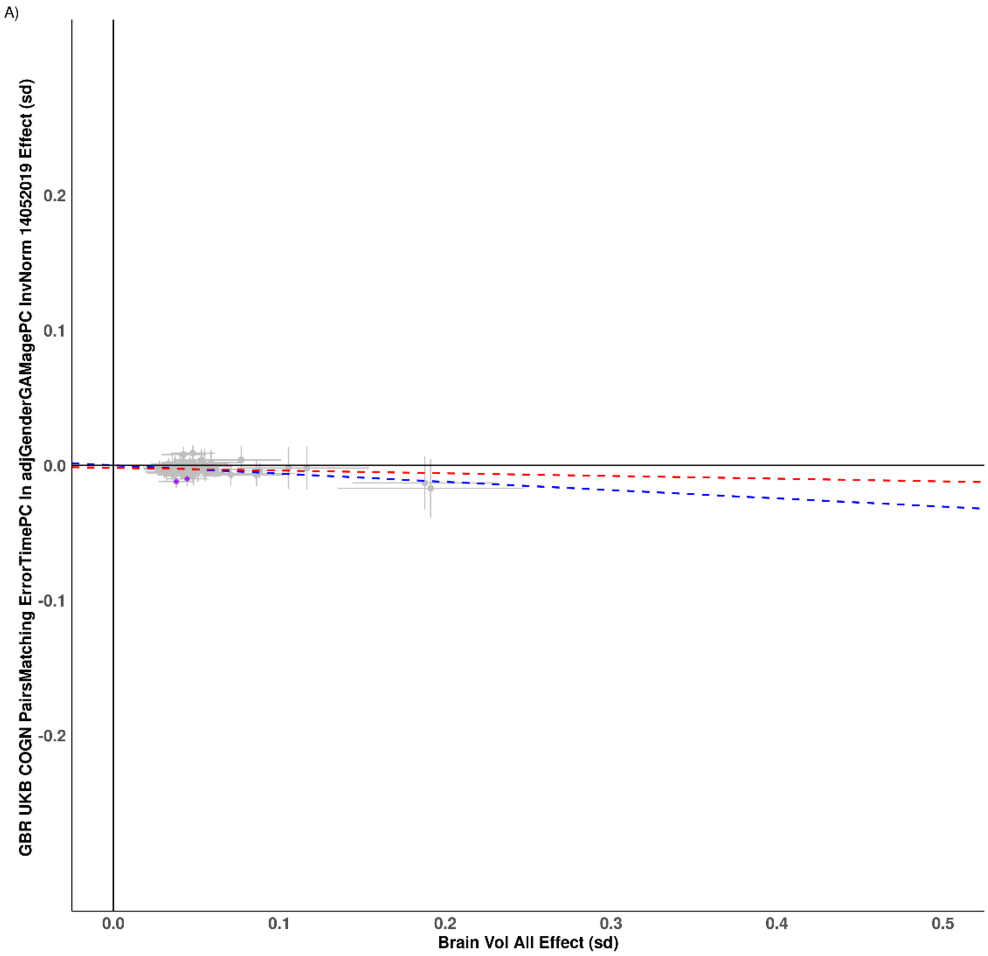

Supplementary figure 42: Effect vs effect plots of variants associated with intracranial volume (*N* = 64) vs pairs matching error time. On *X-axis* are effect size for intracranial volume meta-analysis (Iceland + UKB + ENIGMA + EGGC) and on *y-axis* for pairs matching error time. All effects are plotted for alleles with increasing intracranial volume. Blue line represents slope estimated through inverse variance weighted method (IVW), and red line MR Egger method including intercept. Green dots represent conventional GWAS associations ($P < 5.0\times10^{-8}$), purple are Bonferroni significant associations ($P < 0.05/64 = 0.00078$).

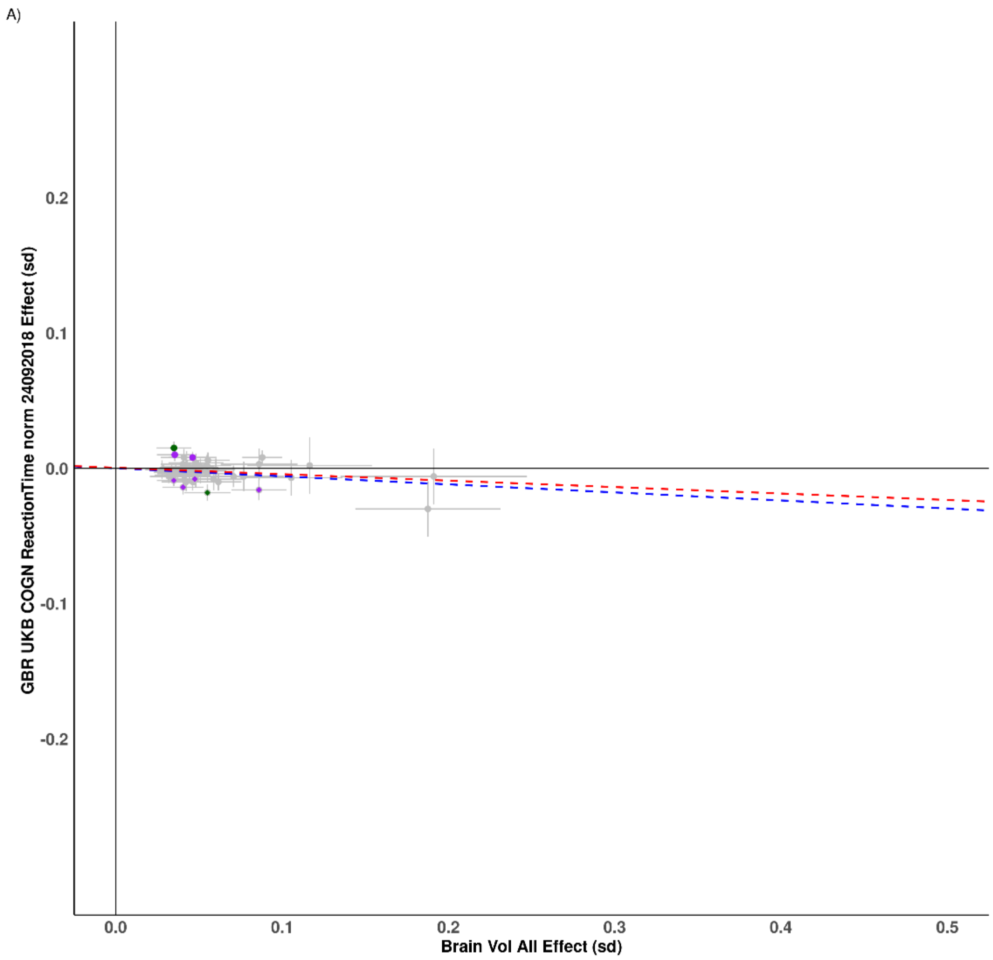

Supplementary figure 43: Effect vs effect plots of variants associated with intracranial volume (*N* = 64) vs reaction time. On *X-axis* are effect size for intracranial volume meta-analysis (Iceland + UKB + ENIGMA + EGGC) and on *y-axis* for reaction time. All effects are plotted for alleles with increasing intracranial volume. Blue line represents slope estimated through inverse variance weighted method (IVW), and red line MR Egger method including intercept. Green dots represent conventional GWAS associations ($P < 5.0\times10^{-8}$), purple are Bonferroni significant associations ($P < 0.05/64 = 0.00078$).

A)

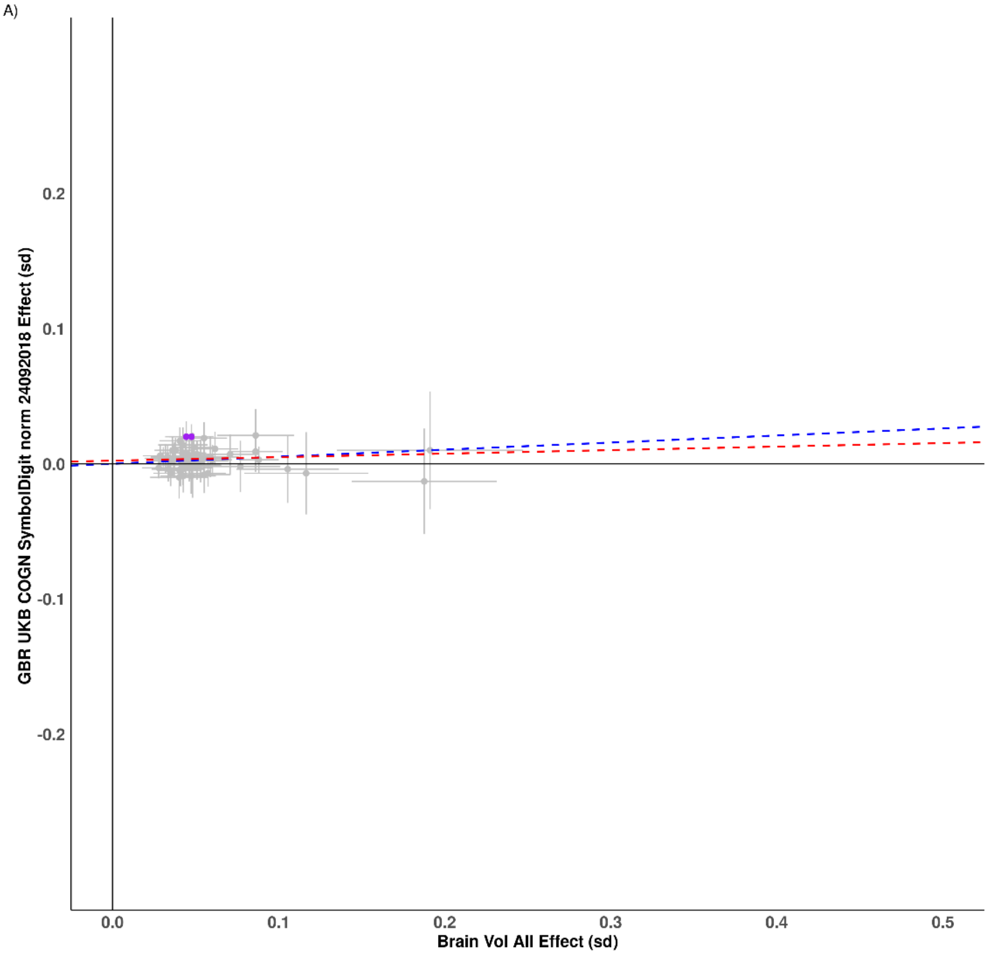

Supplementary figure 44: Effect vs effect plots of variants associated with intracranial volume (*N* = 64) vs symbol digits. On *X-axis* are effect size for intracranial volume meta-analysis (Iceland + UKB + ENIGMA + EGGC) and on *y-axis* for symbol digits. All effects are plotted for alleles with increasing intracranial volume. Blue line represents slope estimated through inverse variance weighted method (IVW), and red line MR Egger method including intercept. Green dots represent conventional GWAS associations ($P < 5.0\times10^{-8}$), purple are Bonferroni significant associations ($P < 0.05/64 = 0.00078$).

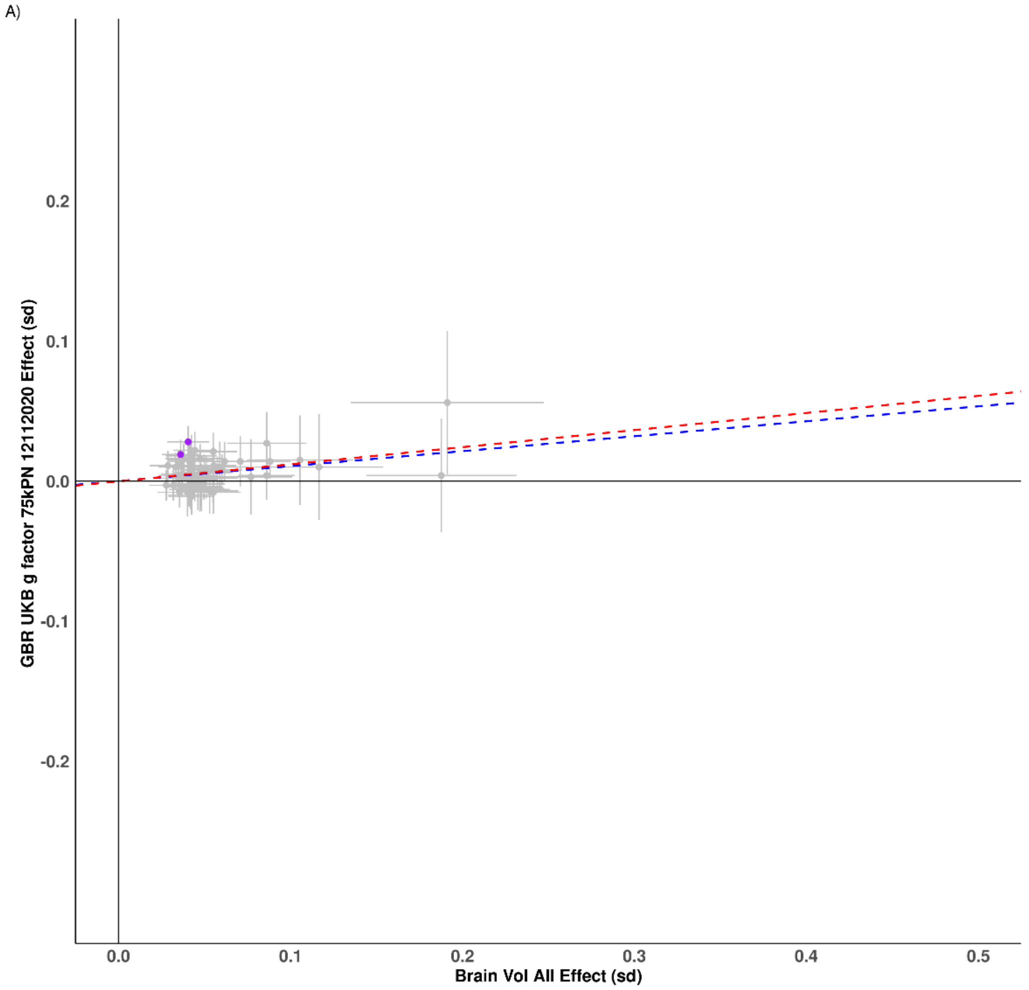

Supplementary figure 45: Effect vs effect plots of variants associated with intracranial volume (*N* = 64) vs g-factor. On *X-axis* are effect size for intracranial volume meta-analysis (Iceland + UKB + ENIGMA + EGGC) and on *y-axis* for g-factor. All effects are plotted for alleles with increasing intracranial volume. Blue line represents slope estimated through inverse variance weighted method (IVW), and red line MR Egger method including intercept. Green dots represent conventional GWAS associations ($P < 5.0\times10^{-8}$), purple are Bonferroni significant associations ($P < 0.05/64 = 0.00078$).

A)

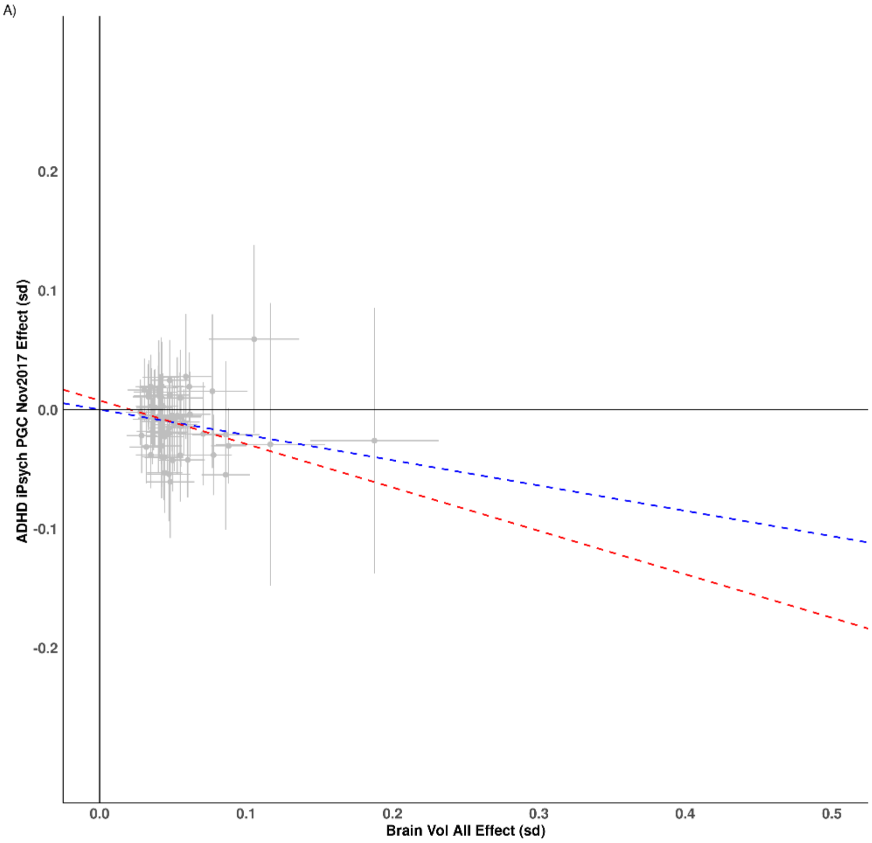

Supplementary figure 46: Effect vs effect plots of variants associated with intracranial volume (*N* = 64) vs ADHD. On *X-axis* are effect size for intracranial volume meta-analysis (Iceland + UKB + ENIGMA + EGGC) and on *y-axis* for ADHD. All effects are plotted for alleles with increasing intracranial volume. Blue line represents slope estimated through inverse variance weighted method (IVW), and red line MR Egger method including intercept. Green dots represent conventional GWAS associations ($P < 5.0\times10^{-8}$), purple are Bonferroni significant associations ($P < 0.05/64 = 0.00078$).

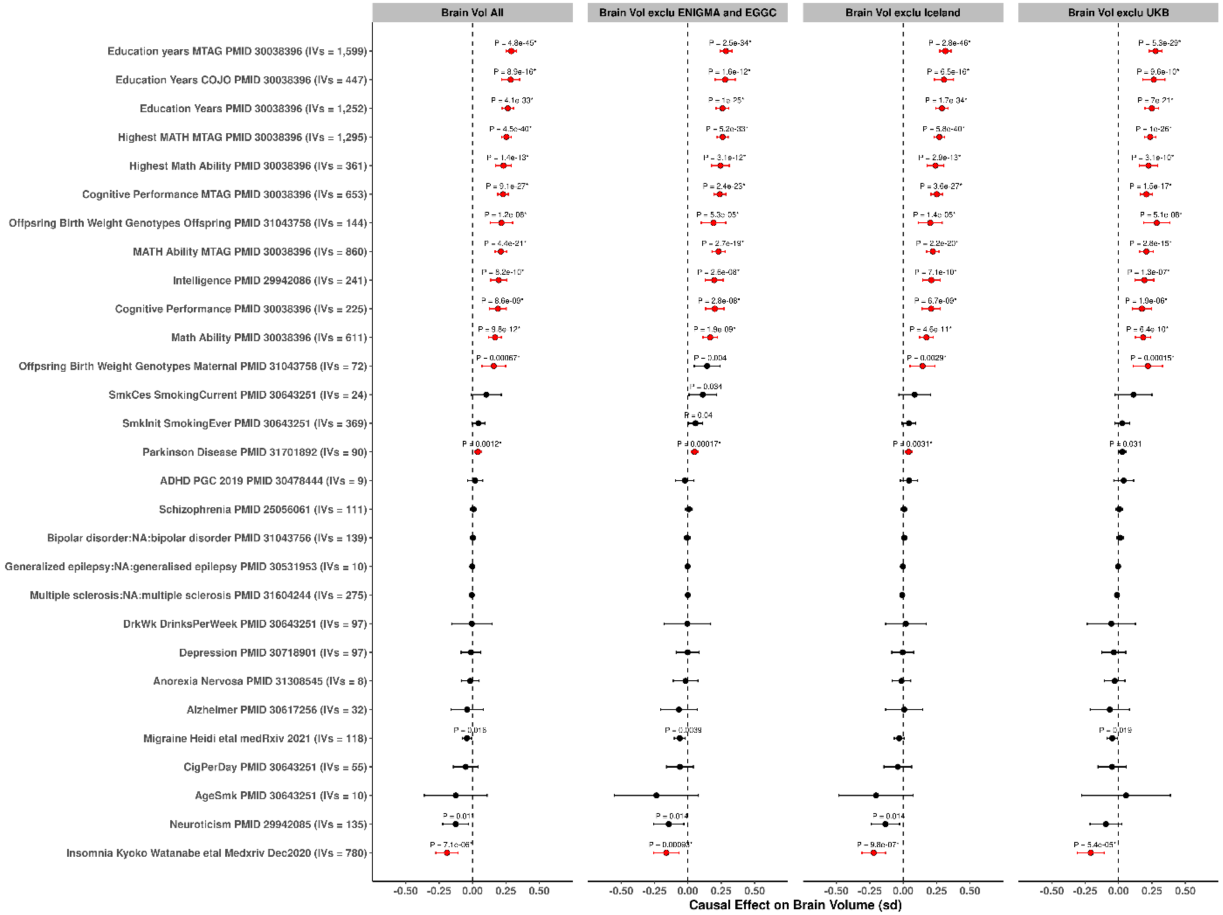

Supplementary Figure 47: Reverse causal association test using instrumental variants from correlated studies for their effect on intracranial volume (A) in full intracranial volume meta-analysis **(B)** intracranial volume excluding ENIGMA/EGGC data (**C**) intracranial volume excluding Iceland data, and **(D)** intracranial volume excluding UK biobank data**.** The analysis was performed using the instrumental variables from studies (y-axis) and their effect sizes on intracranial volume (**Supplementary Table 8c**). IVW (inverse variance weighted) method was used to estimate the causal effect, additionally Egger analysis was performed to detect whether IVW estimates are biased i.e. intercept is different from zero (**Supplementary Table 8d**). The Bonferroni significant associations ($\boldsymbol{P} < 0.05/32 = 1.56\times10^{-3}$) are coloured in red.
