# Supplementary material for: Thirty novel sequence variants impacting human intracranial volume
Source: Brain Commun. 2022 Oct 25;4(6):fcac271. doi: 10.1093/braincomms/fcac271 (PMC9677475; doi:10.1093/braincomms/fcac271)
Supplement: fcac271_Supplementary_Data [file fcac271_supplementary_data.zip › Supplementary_table_legends.pdf]

Supplementary Table legends

Suppl_Table_1

Summary statistics of 64 Intracranial Volume associated variants (30 novel and 34 known associations) in the meta-analysis (N = 79,174).

EA is effect allele, **OA** is other allele, **EAF** is effect allele frequency in percentage**, coding effect** is estimated effect by effect allele.
P is p-value for association, Beta is effect estimate in standard deviation**, Phet** is p-value for heterogenity of meta-analysis**, I2** is phenotypic variance due to heterogenity.
SE is for standard error**, r^2** is a measure of LD with known variant in the loci**, PMID** is for pubmed ID for known variant.
Candidate gene are genes reported based on cis-eQTL, pQTL (cis/trans), and coding variants.
R^2% is the measure of phenotypic variance in percentage explained by respective variant, computed through this **formula: $(2\times EAF\times(1-EAF) \times \beta^2)*100$ (PMID: 31840077)**

Suppl_Table_2a Cis-eQTL analysis of Intracranial Volume associated variants (N=64) using RNA sequence data from deCODE and GTEx v8.

Cis-eQTL analysis of Intracranial Volume associated variants (N=64) using RNA sequence data from deCODE and GTEx v8.
EA is effect allele, **OA** is other allele, **EAF** is effect allele frequency in percentage**, coding effect** is estimated effect by effect allele.
P_{ICV} is p-value for association for Intracranial Volume, $Beta_{ICV}$ is effect estimate in standard deviation for Intracranial Volume.
P_{eQTL} is p-value for association for eQTL from RNA sequence association data, $Beta_{eQTL}$ is effect estimate in standard deviation for respective eQTL.
Tissue in which eQTL is measured, **Source** refers to eQTL data source.
LD friend is top LD of Intracranial Volume variant which is in LD with top eQTL**,** r2 is a measure of LD between Intracranial Volume variant and eQTL variant.
$P_{threshold}$ for eQTL analysis is: 0.05/75,729 = 6.36e-7, where 75,729 independent tests were performed by testing 3,311 genes in 50 tissues.

Suppl_Table_2b pQTL (cis/trans) analysis of Intracranial Volume associated variants (N=64) using SOMAlogic scan of 4,983 protein from plasma of 35,559 Icelanders.

EA is effect allele, **OA** is other allele, **EAF** is effect allele frequency in percentage**, coding effect** is estimated effect by effect allele.
P_{ICV} is p-value for association for Intracranial Volume, B_{etaICV} is effect estimate in standard deviation for Intracranial Volume.
P_{pQTL} is p-value for association for pQTL from protein association data, $Beta_{pQTL}$ is effect estimate in standard deviation for respective pQTL.
Uniport is Uniprot ID for the protein, pos_var is the position of pQTL, TSS_prot is transcription start site of protein
LD friend is top LD of Intracranial Volume variant which is in LD with top eQTL, **r2** is a measure of LD between Intracranial Volume variant and eQTL variant.
Pthreshold for pQTL analysis is: 0.05/4,907/64 = 1.59e-7, where 4,907 aptamers (targetting 4,719 proteins) were tested for 64 ICV variants.

Suppl_Table_3

Summary of PheWAS look up analysis using NHGRI GWAS Catalog34 (https://www.ebi.ac.uk/gwas/)

For PheWAS we used 64 ICV variants (and their LD friends r2 > 0.8) to check whethr are in high LD with reported GWAS marker in NHGRI GWAS.
rsID referes to rs name of the ICV variant, EAF_ICV is effect allele frequency in percentage, P_ICV is pvalue for Intracranial volume association, Beta_ICV is effect size for intracranial volume.
r2 is measure of LD between ICV variant and variant reported in GWAS catalog, distance is base-pair distance between correlated variants, MAPPED_Trait_Category is the category of corresponding trait, MAPPED_TRAIT is corresponding trait, P_VALUE is pvalue of association of corresponding trait from GWAS catalog.

Suppl_Table_4a

Phenotype correlation of ICV, height, and BMI with cortical and subcortical regions (areas, volumes, and thickness) using data from 37,100 UKB participants.

pheno_sMRI is the name of cortical or sub-cortical region trait, **trait** is either ICV/height against which correlation is computed
pearson_corr is measure of Pearson correlation between testes traits, **pearson_p_value** is pvalue for measure of correlation.
intercept is measure of intercept value from simple linear regression of pheno_sMRI vs trait, **slope** is measure of slope showing relationhsip between tested traits from linear regression, **p_value** is p value of association from linear regression.

Suppl_Table_4b

Intracranial volume associated variants (N=64) tested for association with volumes of cortical and sub-cortical regions, all adjusted for ICV,(*N*=115).

EA is effect allele, **OA** is other allele, **EAF** is effect allele frequency in percentage**, coding effect** is estimated effect by effect allele.
$\boldsymbol{P}_{Intracranial\_volume}$ is p-value for association for Intracranial volume, $\mathbf{Beta}_{\mathrm{Intracranial\_volume}}$ is effect estimate in standard deviation for Intracranial volume.
P_sMRI is p-value for association for respective sMRI region, **Beta_sMRI** is effect estimate in standard deviation for respective sMRI region.
Significance threshold adjusted for multiple testing (*P* <0.05/115/64 =6.8e-6)

Suppl_Table_5

MAGMA based genes set/pathway enrichment analysis using Intracranial Volume gwas meta-analysis.

mSignatureDB version 7.4 was used to reterive annotated genes/pathway sets.
mSignatureDB.Pathway_Source corresponds to genes-set data from molecular signature data base. URL: https://www.gsea-msigdb.org/gsea/msigdb/.
P is pvalue for association from MAGMA gene set analysis, **number of genes** represent genes overlap by pathway/genes-set term, .

Bonf. $\boldsymbol{P}_{\mathrm{threshold}}$ for the number of tests is **0.05/9,753 = 5.13e-6**

Suppl_Table_6a

Phenome-wide bivariate genetic correlation of Intracranial Volume vs published studies estimated through LDSC.

Phenotype is the name of tested phenotype, **Phenotype.Domain** is the categerized domain of respective phenotype, **Reference** contains pubmed-ID of respective GWAS if available.
rg is measure of genetic correlation (rg is not a bounded measure and in case of sample overlap, the LDSC may estimate this beyound -1/+1. **SE** is standard error measure of rg.
Z is estimate z-score for rg measure, and ***P*** I p-value for rg measure.
Only results shown where ***rg*** measure is significant for multiple testing and is insensitive to sample overlap.
P threshold < 0.05/1483 = 3.4e-5 and in leave one sample out analysis **P < 0.05/94 = 5.32e-4**

Suppl_Table_6b
Phenotypic correlation of ICV/HC with neurological phenotypes, personality and cognitive/learning traits.

Suppl_Table_7

Summary of Intracranial Volume polygenic risk score tested in Iceland and UKB.

Predictor refers to source GWAS used for PRS while **Tested in** refers to population in which PRS is tested.
WT refers weight used in PRS (roughly translates to p-value threshold used to compute PRS).
YOB_RANGE column refers to year of birth information used for participants.
R2_raw% is variance explained in percentage by model exlcuding PRS.
R2_delta% is phenotypic variance explained by PRS.
P is p-value for association, **Beta** is estimated effect by PRS, and **SE** is for standard error.

Suppl_Table_8a

Mendelian randomization analysis using Intracranial Volume variants as an instrumental variables.

rsID is marker ID, **marker** is chr:pos_hg38_OA_EA.
$\mathbf{P_{ICV}}$ is p-value for association for Intracranial Volume given in column 'H', $\mathbf{Beta_{ICV}}$ is effect estimate in standard deviation for Intracranial Volume given in column 'H'.
$\boldsymbol{P\_{pheno2}}$ is p-value for association of Pheno2 given in column 'I' , **Beta_pheno2** is effect estimate in standard deviation of Pheno2 given in column 'I' .
Reference is PMID/reference of the Pheno2.
EAF is effect allele frequency.
References for **outcome.pheno** are avaiable in **Supplementary Table 8b**.

Suppl_Table_8b

Input data of Intracranial Volume variants (Instrumental variables) and respective outcome trait used for MR analysis in Suppl Table 8a.

Exposure.phenotype is name of the tested Intracranial Volume GWAS meta-analysis used as exposure trait in MR analysis.
Outcome.Phenotype is name of phenotype from which GWAS data was used for Intracranial Volume variants as an outcom.phenotype.

N_{IV} is number of Intracranial Volume variants used as instrumental variables.
CasualEffect is estimated causal effect, **SE** is standard error in estimated effect, ***P*** is p-value for association test, **LCI** is estimated lower confidence interval, **UCI** is for upper confidence interval.
Sample.Overlap: description about whether we observed sample overlap in exposure and outcome GWAS SS data.
IVW refers to Inverse-variance-weighted method, **MR_Egger refers to MR_Egger** method used to measure pleiotropy i.e. whether intercept '**MR_Egger_intercept**' is different from zero.

Suppl_Table_8c

Input data of respective exposure trait (as Instrumental variables) and ouctome Intracranial Volume data used for MR analysis in Suppl Table 8d.

Input data of respective exposure trait (as Instrumental variables) and ouctome Intracranial Volume data used for MR analysis in Suppl Table 8d.
rsID is marker ID, **marker** is chr:pos_hg38_OA_EA.
P_{ICV} is p-value for association for Intracranial Volume given in column 'I', **$Beta_{ICV}$** is effect estimate in standard deviation for Intracranial Volume given in column 'I'.
P_exposure is p-value for association of exposure given in column 'G' , **Beta_expore** is effect estimate in standard deviation of exposure given in column 'G' .
Reference is PMID/reference of the Pheno2.
EAF is effect allele frequency.
References for **outcome.pheno** are available in **Supplementary Table 8b**.

Suppl_Table_8d

Mendelian randomization analysis using Intracranial Volume variants as an outcome trait.

Input data of exposure (Instrumental variables) and BV trait used for MR analysis in Suppl Table 8c.
Exposure.phenotype is name of the tested Intracranial_Volume GWAS meta-analysis used as exposure trait in MR analysis.
Outcome.Phenotype is name of phenotype from which GWAS data was used for Intracranial_Volume variants as an outcome phenotype.

N_{IV} is number of Intracranial_Volume variants used as instrumental variables.
CasualEffect is estimated causal effect, **SE** is standard error in estimated effect, ***P*** is p-value for association test, **LCI** is estimated lower confidence interval, **UCI** is for upper confidence interval.
Sample.Overlap: description about whether we observed sample overlap in exposure and outcome GWAS SS data.
IVW refers to Inverse-variance-weighted regression method, **MR Egger refers to Egger** regression method used to measure pleiotropy i.e. whether intercept '**MR_Egger_intercept**' is different from zero.
